# Integrating single-cell RNA sequencing with spatial transcriptomics reveals immune landscape for interstitial cystitis

**DOI:** 10.1038/s41392-022-00962-8

**Published:** 2022-05-20

**Authors:** Liao Peng, Xi Jin, Bo-ya Li, Xiao Zeng, Bang-hua Liao, Tao Jin, Jia-wei Chen, Xiao-shuai Gao, Wei Wang, Qing He, Guo Chen, Li-Na Gong, Hong Shen, Kun-jie Wang, Hong Li, De-yi Luo

**Affiliations:** Department of Urology, Institute of Urology, West China Hospital, Sichuan University, Chengdu, Sichuan P.R. China

**Keywords:** Urogenital diseases, Genome informatics

## Abstract

Interstitial cystitis (IC) is a severely debilitating and chronic disorder with unclear etiology and pathophysiology, which makes the diagnosis difficult and treatment challenging. To investigate the role of immunity in IC bladders, we sequenced 135,091 CD45^+^ immune cells from 15 female patients with IC and 9 controls with stress urinary incontinence using single-cell RNA sequencing (scRNA-seq). 22 immune subpopulations were identified in the constructed landscape. Among them, M2-like macrophages, inflammatory CD14^+^ macrophages, and conventional dendritic cells had the most communications with other immune cells. Then, a significant increase of central memory CD4^+^ T cells, regulatory T cells, GZMK^+^CD8^+^ T cells, activated B cells, un-switched memory B cells, and neutrophils, and a significant decrease of CD8^+^ effector T cells, Th17 cells, follicular helper T cells, switched memory B cells, transitional B cells, and macrophages were noted in IC bladders. The enrichment analysis identified a virus-related response during the dynamic change of cell proportion, furthermore, the human polyomavirus-2 was detected with a positive rate of 95% in urine of patients with IC. By integrating the results of scRNA-seq with spatial transcriptomics, we found nearly all immune subpopulations were enriched in the urothelial region or located close to fibroblasts in IC bladders, but they were discovered around urothelium and smooth muscle cells in control bladders. These findings depict the immune landscape for IC and might provide valuable insights into the pathophysiology of IC.

## Introduction

Interstitial cystitis (IC), referred to bladder pain syndrome, characterized by intense pelvic pain and urinary symptoms, is a severely debilitating and chronic disorder.^1^ The incidence of IC ranges from 0.01 to 6.5% with about five times more diagnosed females than the males.^2^ The etiology and pathophysiology of IC still remain an enigma, which makes the diagnosis difficult and treatment challenging.^1^ There are great chances of misdiagnosis or underdiagnosis, and it usually takes 2–11 years to get an accurate diagnosis. The current therapies of IC showed limited effects and relatively high recurrence rates in the long-term follow-up.^3^ About 10% of the diagnosed patients have to receive destructive surgeries (augmentation ileocystoplasty, urinary diversion, etc.) followed by stepwise therapeutic approaches, and 20% of whom have to face the failures.^4^ Therefore, there is a pressing need to understand the molecular mechanisms underlying the IC development and to identify more efficient targets for therapeutic treatments.

There have been several theories suggesting the causes of IC, including inflammation, neural changes, defects in the wall of bladder, and activated mast cells, but none of which has fully explained the manifestations of this disease.^5^ Although no one could determine whether IC is an autoimmune disease, the current evidence shows that immunity might play an important role in the progress of IC.^6–11^ Abnormal immunity is a well-known histological feature of IC.^9^ Thus, we aimed to investigate the immune landscape (the distribution of immune cell subsets) in IC bladders and the specific stage in which the immunity might involve in the progress of IC. The previous studies investigating the molecular pathways of IC focused primarily on the unbiased analysis of bladder tissue, blood, and urine through flow cytometry, pathological features characterization, as well as microarray gene expression.^12–16^ To what extent they can reflect the inflammatory conditions, cell subpopulations, and immune states is still unknown.

Single-cell RNA sequencing (scRNA-Seq), with the ability to reveal distinct subpopulations among cell types, has emerged as a powerful tool to capture the complex profiles of immune infiltrations in diseases.^17^ The method combining scRNA-seq with mass cytometry (cytometry by time-of-flight (CyTOF)) has been used to define inflammatory cell states in rheumatoid arthritis joint synovial tissues.^18^ Spatial transcriptomics (ST) is currently an evident method to identify cell types and gene expression levels on a structural and spatial level in selected tissue.^19,20^ Integrating scRNA-seq with ST has been applied to uncover the tissue architecture in pancreatic ductal adenocarcinomas.^21^ To better understand the role of immunity in IC bladders, we first conducted a comprehensive phenotypic and functional investigation of immune parameters (using only CD45^+^ single cells) through scRNA-Seq combining CyTOF. Then, the immune views resulted from scRNA-Seq were integrated with the results of ST through multimodal intersection analysis. The findings of this study reveal the immune landscape of bladder in IC patients and may pave the way for future studies of pathophysiology and therapy.

## Results

### Single-cell transcriptional profile of CD45^+^ immune cells in IC bladder

**Fig. 1:**
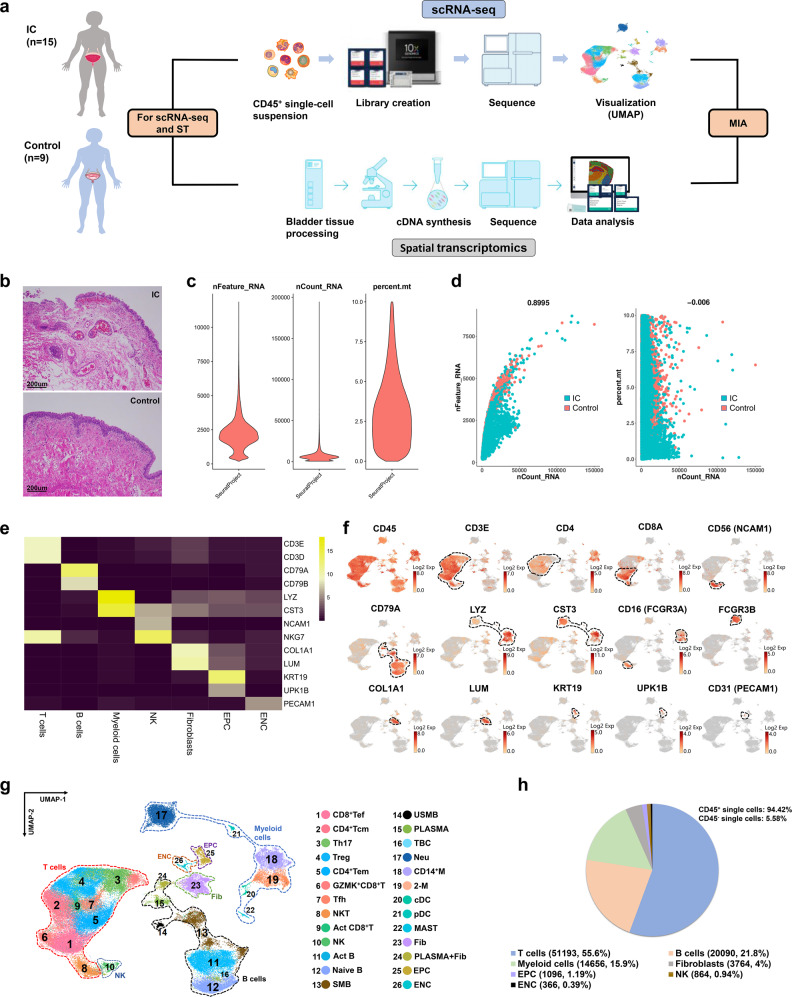
Overview of workflow for single-cell RNA-sequence (scRNA-seq) and spatial transcriptomics (ST). **a** CD45^+^ single cells from IC (*n* = 15) and control (*n* = 9) bladders were collected to construct an immune landscape. ST views were established based on 3 IC bladders and 3 control bladders. The constructed atlases were combined via multimodal intersection analysis. **b** Pathological features of bladders of IC patients and controls were presented by H&E images (bar = 200 μm). **c** Violin plots showing the quality control of single-cell data. **d** A high correlation coefficient of 0.8995 between cell counts and genes was observed, but not in cell counts and mitochondrial genes. **e** Canonical marker genes for immune cell subsets. **f** The expression of selected marker genes in the immune landscape. **g** UMAP plot presenting the immune landscape of IC bladders. **h** The proportion of each cell type. IC interstitial cystitis, UMAP uniform manifold approximation and projection, CD8^+^ Tef CD8^+^ effector T cell, CD4^+^ Tcm central memory CD4^+^ T cell, Treg regulatory T cell, CD4^+^ Tem effector memory CD4^+^ T cell, Tfh follicular helper T cell, Act CD8^+^ T activated CD8^+^ T cell, NK CD56^bright^ CD16^low^ natural killer cell, Act B activated B cell, SMB switched memory B cell, USMB un-switched memory B, PLASMA plasma cell, TBC transitional B cell, Neu neutrophil, CD14^+^ M inflammatory CD14^+^ macrophage, 2-M M2-like macrophage, cDC conventional dendritic cell, pDC plasmacytoid dendritic cell, MAST mast cell, Fib fibroblast, EPC epithelial cell, ENC endothelial cell

To decipher and construct an immune landscape for IC, scRNA-seq was applied to examine the transcriptomic heterogeneity that is concealed in population-averaged measurements (Fig. 1a). 15 IC patients and 9 controls were included as the inclusion and exclusion criteria, respectively (Tables S1 and S2). The characteristic pathological findings in the bladder wall of IC were identified by cystoscopy (Supplementary Fig. 1a). The pathological features showed that the denudation or anatomical loss of urothelium, angiogenesis, and immune cell infiltration are common in IC bladders compared with the controls (Fig. 1b). Bladder specimens from patients with IC and controls were freshly collected, dissected, and digested into single cells (Table S3). A total number of 135,091 CD45^+^ immune single cells were isolated and sequenced from the 24 samples (Table S4). In all, 92,029 high-quality CD45^+^ immune cells were further analyzed after strict quality control (Fig. 1c). A high correlation coefficient between the cell counts and genes was calculated (*R* = 0.8995) but not observed between the cell counts and mitochondrial genes (*R* = −0.006) (Fig. 1d). The dimension reduction was performed through principal component analysis (PCA), followed by visualization conducted using uniform manifold approximation and projection (UMAP) method. Seven cell types with the most distinguishable hematopoietic and histionic lineage-defining genes were detected, including T cells: CD3E (51,193, 55.6%), B cells: CD79A (20,090, 21.8%), myeloid cells: LYZ and CST3 (14,656, 15.9%), fibroblasts: COL1A1 and LUM (3764, 4%), epithelial cells: KRT19 and UPK1B (1096, 1.19%), NK cells: CD56/NCAM1, CD16/FCGR3A, and NKG7 (864, 0.94%), and endothelial cells: CD31/PECAM1 (366, 0.39%) (Fig. 1e–h). It was noted that the current cell landscape was composed of 94.42% of CD45^+^ immune cells and 5.58% of CD45^−^ single cells after removal of doublets (Fig. 1h). The detected immune cells in IC bladders including T cells, B cells, plasma cells, macrophages, neutrophils, and mast cells were similar to the findings in previous studies.^8,9,11^ However, further classified cell types such as regulatory T cells (Treg), natural killer T cells (NKT), CD56^bright^ CD16^low^ NK cells, switched/un-switch memory B cells (SMB/USMB), M2-like macrophages, as well as dendritic cells (DC) were rarely reported. With the identification of these cells, a more complicated and complete immune map could be pictured in patients with IC.

### Stepwise clustering of the T and B cells lineages found the dysfunction of CD8^+^ T cells and abnormal activation of B cells

**Fig. 2:**
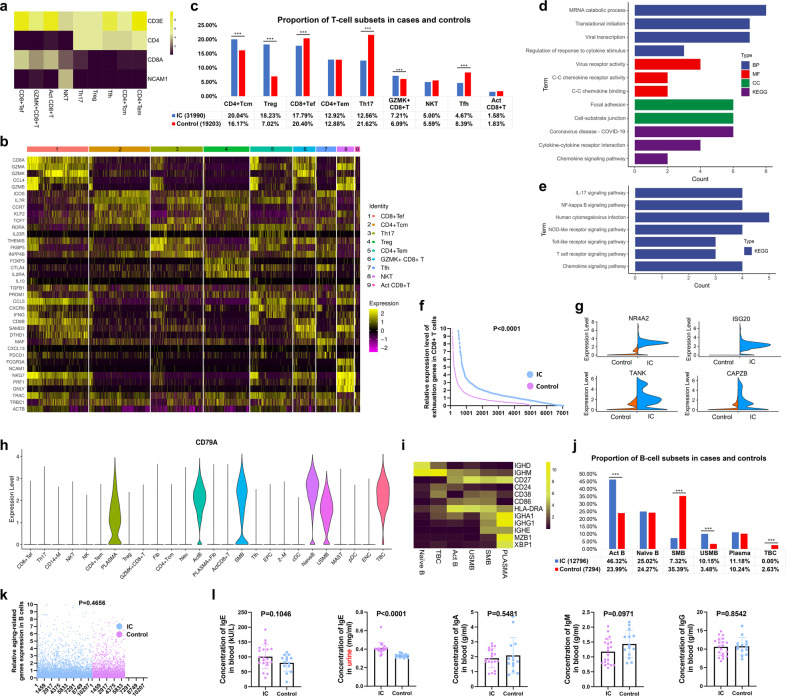
Focused analysis of T and B cells. **a** The expression of CD3E, CD4, CD8A, and NCAM1 across the clusters. **b** Heatmap showing the expression of selected marker genes across the T cell clusters. **c** The changes of proportion in T cell subsets between the two groups (***P < 0.001). **d** Bar chart showing the significant GO and KEGG terms of Tregs in the IC group compared with the control group. **e** Significant KEGG terms of Th17 in the IC group compared with the control group. **f** The relative expression level of all exhausted genes in CD8^+^ T cells between the IC and control groups. **g** The expression level of representative exhausted markers in the two groups. **h** Violin plot showing the expression of B cell marker gene (CD79A) across the clusters. **i** Heatmap showing the expression of selected marker genes across the B cell clusters. **j** The changes of proportion in B cell subsets between the two groups (***P < 0.001). **k** The relative expression level of aging-related genes in B cells between the IC and control groups. **l** The expression leve**l** of immunoglobulins including IgA, IgM, IgG, and IgE in plasma or urine of IC patients and controls (mean ± standard deviation, Mann–Whitney *U*-test, two-tailed; IC, *n* = 20, control, *n* = 14). IC interstitial cystitis, CD8^+^ Tef CD8^+^ effector T cell, CD4^+^ Tcm central memory CD4^+^ T cell, Treg regulatory T cell, CD4^+^ Tem effector memory CD4^+^ T cell, Tfh follicular helper T cell, Act CD8^+^ T activated CD8^+^ T cell, NK CD56^bright^ CD16^low^ natural killer cell, Act B activated B cell, SMB switched memory B cell, USMB un-switched memory B, PLASMA plasma cell, TBC transitional B cell

To characterize the changes in individual T cell subsets between the IC and control groups, we subclustered T cells and obtained 9 subsets according to the expression of the canonical T cell markers. We identified 5 clusters of CD4^+^ T cells, 3 clusters of CD8^+^ T cells, and 1 cluster of NKT cells (Fig. 2a).

Within the CD4^+^ T cell compartment, as shown in Fig. 2b, two helper T cell clusters including Tregs (FOXP3, CTLA4, and IL-2RA) and follicular helper T cells (Tfh) (MAF, CXCL13, ICOS, and PDCD1) were identified by expression of their unique transcription factors and markers. We found IL-10, an essential anti-inflammatory cytokine,^22^ was nearly undetectable in Tregs, which may imply the disorder of immunomodulation in the IC group. We then observed a T helper 17 cell (Th17) cluster that upregulated RORA gene that is a specific transcription factor of Th17, making them Th17 cells. We subsequently identified a CD3D^+^ T cell cluster marked by high expression of genes typically associated with NK cell activity (e.g., NCAM1, FCGR3A, PRF1, NKG7, and GNLY), making them NKT cells. Another cluster was defined as CD56^bright^ CD16^−^ NK cells with the gene profiles including CD16/FCGR3A^-^ NCAM1^+^ TYROBP^+^ KIT^+^ TCF7^+^ IL-7R^+^ RUNX2^+^ PRF1^low^ and GZMB^low^ (Table S5). Finally, the effector memory CD4^+^ T cells (CD4^+^ Tem) were recognized by their high expression of CCL5, CXCR6, and PRDM1, and the central memory CD4^+^ T cells (CD4^+^ Tcm) were identified by their expression of IL-7R, CCR7, and SELL. The CD69 and ICOS were upregulated in these clusters suggesting that these cells were more likely to be CD4^+^ Tcm rather than naive T cells. The expression of TCF7 and KLF2 indicated an early central memory T cell phenotype^23^ (Fig. 2b).

Analyses on the proportion of T cell subsets demonstrated a significant increase of Tregs and a decrease of Th17 cells in IC bladders (Fig. 2c). Gene ontology (GO) and Kyoto Encyclopedia of Genes and Genomes (KEGG) enrichment analysis of differentially expressed genes (DEGs) in the IC group compared with the control group were performed. DEGs of Tregs were mainly enriched in mRNA catabolic process, virus receptor, and focal adhesion (Fig. 2d). However, DEGs of Th17 cells were found mainly enriched in classical inflammatory signaling pathways, such as IL-17 signaling pathway and NF-kappa B signaling pathway (Fig. 2e).

With high expression levels of CD8A and cytotoxic genes (GZMA, GZMK, GZMB, NKG7, and PRF1), two CD8^+^ effector T cell (CD8^+^ Tef) clusters were identified. Cells belong to CD8^+^ Tef highly expressed cytotoxic markers, and the cells in GZMK^+^ CD8^+^ T cell cluster had the highest level of GZMK instead of GZMA or GZMB. The activated CD8^+^ T cells (Act CD8^+^ T) were defined with the expression of activated genes such as TRAC, TRBC1, and ACTB, but it was with a relatively lower level of cytotoxic activity (Fig. 2a, b). The exhausted CD8^+^ T cells in chronic infections have limited effector function.^24^ The CD8^+^ T exhaustion in our study was noted by the expression of exhausted markers such as NR4A2, ISG20, TANK, and CAPZB in three CD8^+^ T clusters (Supplementary Fig. 1b, c). As expected, the expression level of exhausted genes in the IC group was relatively higher than that in the control group (Fig. 2f, g), suggesting the dysfunction of CD8^+^ T in this chronic disorder.

To further investigate the transcriptional changes in the B cell subpopulations, we subclustered B cells and found 6 B cell subsets (Fig. 2h), including naive B cells (CD27^−^ IGHM^+^ IGHD^+^), transitional B cell (TBCs) (CD27^−^ CD24^+^ IGHM^low^ IGHD^low^), activated B cells (CD27^+^ CD86^+^ HLA-DRA^+^), un-switched memory B cells (USMBs) (CD27^+^ CD38^+^ IGHM^low^ IGHD^low^), switched memory B cells (SMBs) (CD27^+^ IGHM^-^ IGHA1^+^ IGHG1^+^ IGHE^+^), and plasma cells (MZB1^+^ XBP1^+^) (Fig. 2i). Additionally, we detected a cluster containing fibroblasts (DCN, FBLN1, and COL1A1) and plasma cells (IGHA1, MZB1, and XBP1) (Supplementary Fig. 1d). This cluster was then divided into distinct cell types for downstream analysis.

The significant increase of activated B cells and USMBs, and the decrease of SMBs and TBCs were noticed (Fig. 2j). Most of the B cells in the IC group were activated B cells (46.32%), suggesting that the B cell activation became increasingly intense in the IC group. Although a gene expression signature that could indicate age-associated B cells was detected (Supplementary Fig. 1e), the expression level of aging-related genes was not significantly different between the IC and control groups (Fig. 2k and Supplementary Fig. 1f). The abnormal activation of B cells and identification of plasma cells implied that IC patients might undergo a robust humoral immune response. Therefore, the immunoglobulins including IgA, IgM, IgG, and IgE from the plasma and urine of the patients and controls were detected. Although there was no statistical difference between the two groups regarding all immunoglobulins in plasma, a significant increase of IgE in the urine of IC patients was noticed (Fig. 2l), suggesting IgE as a potential urine biomarker for IC.

### Classification and annotation of myeloid-cell clusters revealed immune-related macrophages

**Fig. 3:**
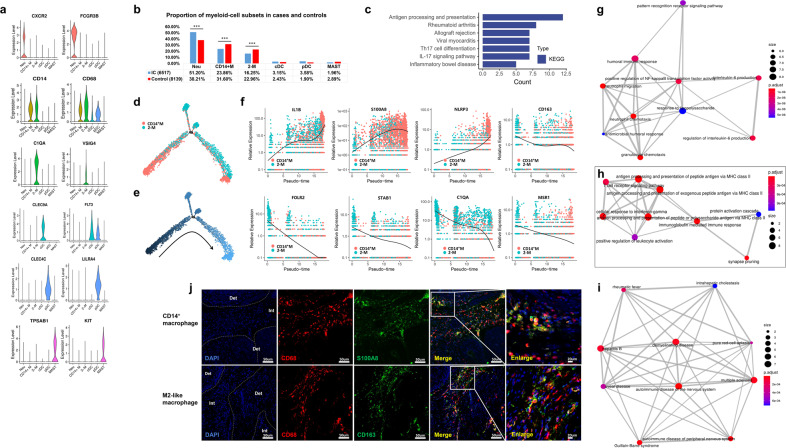
Focused analysis of myeloid cells. **a** Violin plots showing the expression level of selected marker genes across the clusters. **b** The changes of proportion in myeloid-cell subsets between the two groups (***P < 0.001). **c** Significant GO and KEGG terms of neutrophils in the IC group compared with the control group. **d**, **e** Pseudotime analysis depicted the developmental trajectory from CD14^+^ macrophages to M2-like macrophages. **f** Scatter plots showing the expression changes of inflammatory genes (IL-1β, S100A8, and NLRP3) and phagocytosis genes (CD163, FOLR2, STAB1, C1QA, and MSR1) from CD14^+^ macrophages to M2-like macrophages over time. **g** Functional analysis of DEGs of inflammatory CD14^+^ macrophages**. h** Functional analysis of DEGs of M2-like macrophages**. i** Disease enrichment analysis of DEGs of M2-like macrophages. **j** Immunofluorescence staining of CD68, S100A8, and CD163 on frozen sections (Det detrusor, Int interstitial layer; IC, *n* = 15; control, *n* = 9). IC interstitial cystitis, DEGs differentially expressed genes

We then delineated the molecular and functional differences in myeloid cells. Six cells types were identified, including neutrophils (CXCR2 and FCGR3B), inflammatory CD14^+^ macrophages (CD14 and CD68), M2-like macrophages (C1QA and VSIG4), conventional dendritic cell (cDC) (CLEC9A and FLT3), plasmacytoid dendritic cell (pDC) (CLEC4C and LILRA4), and mast cells (TPSAB1 and KIT) (Fig. 3a). Most of the myeloid cells in IC bladders were neutrophils (51.20%) instead of mast cells (Fig. 3b). The number of mast cells in IC bladders is small, and their role is unclear.^25^ KEGG analysis of DEGs in neutrophils in the IC group compared with the control group showed significant enrichments took place in antigen processing and presentation and rheumatoid arthritis pathways (Fig. 3c). Pseudotime analysis depicted the mainstream developmental trajectory from CD14^+^ macrophages to M2-like macrophages (Fig. 3d, e). In the process of differentiation, pro-inflammatory genes (IL-1β, S100A8, and NLRP3) were upregulated in CD14^+^ macrophages but gradually downregulated over time. Meanwhile, phagocytic genes and regulatory receptors (CD163, FOLR2, STAB1, C1QA, and MSR1) were gradually upregulated in M2-like macrophages (Fig. 3f). Compared with the control, the function of inflammatory CD14^+^ macrophages was related to inflammation production, such as IL-6 production and NF-kappa B signaling regulation (Fig. 3g). However, M2-like macrophages mainly modulated immunity via MHC class II molecule (Fig. 3h). Moreover, DEGs of M2-like macrophages were mainly enriched in autoimmune disorders such as multiple sclerosis and Guillain-Barre syndrome (Fig. 3i). Macrophage polarization and M1/M2 balance play an important role in anti-microbial defense, asthma and allergy, fibrosis, wound healing, and autoimmunity.^26^ To validate the existence of two different macrophages, immunofluorescence staining of CD68, S100A8, and CD163 was performed (Fig. 3j).

### Confirmation of the immune phenotypical heterogeneity by CyTOF

**Fig. 4:**
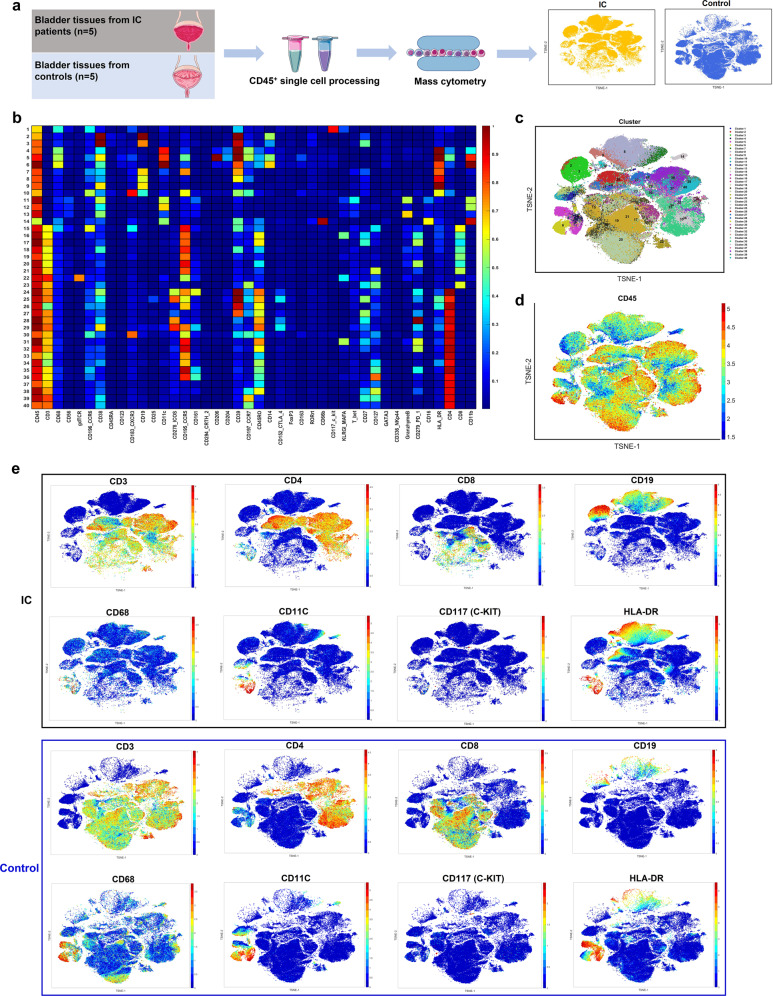
Mass cytometry (CyTOF) confirmed the phenotypical heterogeneity of immune cells in IC bladders. **a** Overview of workflow for CyTOF (IC, *n* = 5; control, *n* = 5). **b** The heatmap showed the relative protein expression level of selected markers in bladder tissues, and generated clusters are visualized by T-SNE plots (**c**). **d** Feature plots showing the expression of the canonical marker gene (CD45) across immune cells. **e** The selected markers of T cell (CD3), CD4^+^ T cell (CD4), CD8^+^T cells (CD8), B cell (CD19), macrophage (CD68), dendric cell (CD11C), mast cell (C-KIT), and activated gene (HLA-DR) were detected in the IC and control groups. IC interstitial cystitis

CyTOF was performed to characterize immune subpopulations within bladders in patients with IC, as well as to validate the scRNA-seq results (Fig. 4a). The protein level of selected markers in each cluster is shown in Fig. 4b. Forty clusters from the IC patients and controls were identified in the construed map (Fig. 4b, c). CD45^+^ immune cells were captured to performed CyTOF analysis (Fig. 4d). According to the canonical markers in Table S6, distinct cell subsets including CD4^+^ T cells (CD3^+^CD4^+^), CD8^+^ T cells (CD3^+^CD8^+^), B cells (CD19), macrophages (CD68), DCs (CD11C), mast cells (KIT), and activated cells (HLA-DR) were identified and the putative cell types were in line with the results of scRNA-seq (Fig. 4e). Importantly, the changes of major cell proportions were accordant with the results of scRNA-seq (Supplementary Fig. 2). Together with the scRNA-seq-based definition of clusters, the data helped validate and build a cellular immune cell atlas in IC bladders.

### Immune activation network of IC

**Fig. 5:**
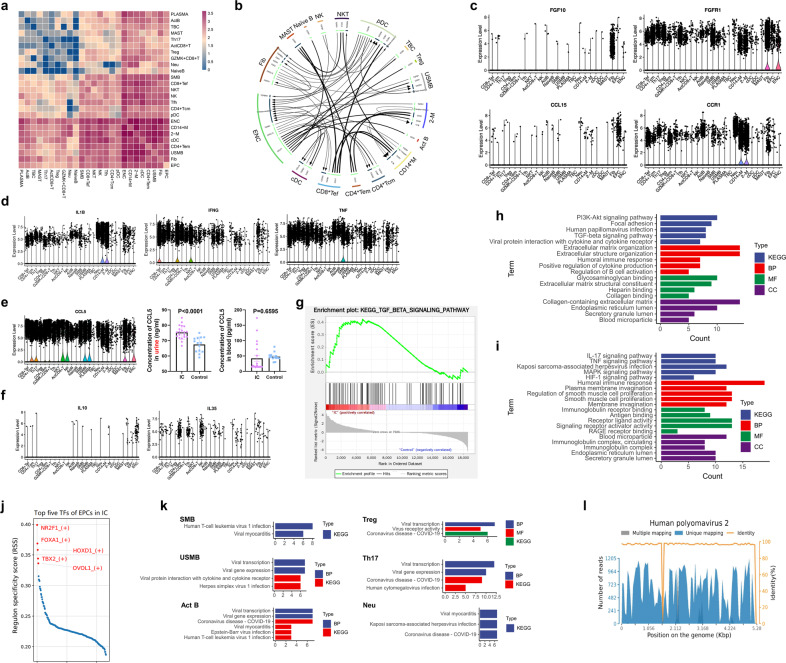
Cellphone DB analysis based on scRNA-seq showed the crosstalk in the immune network. **a** Heatmap showing the distribution of interaction pairs across the cell types. **b** Cell–cell communication network in IC based on the top 50 interaction pairs. **c** Violin plot showing the expression of interaction pairs including CCL15-CCR1 on epithelial cells and FGF10-FCGR1 on fibroblasts. **d** The expression of inflammatory genes including TNF, IL-1β, and IFNG across the clusters. **e** Violin plot showed the upregulated expression of CCL5 across immune cells, which was validated by Enzyme-linked-immunosorbent serologic assay tests in urine (*P* < 0.0001), but not in blood (*P* = 0.6595) (Mann–Whitney *U*-test, two-tailed; IC, *n* = 20; control, *n* = 14). **f** The downregulated expression level of IL-10 and IL-35 across immune cells. **g** The differentially expressed genes (DEGs) in Tregs in the IC group compared with the control group were enriched and upregulated in TGF-β signaling pathway. **h** Enrichment analysis of DEGs of epithelial cells. **i** Enrichment analysis of DEGs of fibroblasts. **j** Top 5 transcription factors of epithelial cells. **k** Bar plot showing the virus-related enriched results in selected cell types. **l** Human po**l**yomavirus-2 was detected in IC urine with 95% positive rates, but 0% in control urine (IC, *n* = 20; control, *n* = 14). IC interstitial cystitis, CD8^+^ Tef CD8^+^ effector T cell, CD4^+^ Tcm central memory CD4^+^ T cell, Treg regulatory T cell, CD4^+^ Tem effector memory CD4^+^ T cell, Tfh follicular helper T cell, Act CD8^+^ T activated CD8^+^ T cell, NK CD56^bright^ CD16^low^ natural killer cell, Act B activated B cell, SMB switched memory B cell, USMB un-switched memory B, PLASMA plasma cell, TBC transitional B cell, Neu neutrophil, CD14^+^ M inflammatory CD14^+^ macrophage, 2-M M2-like macrophage, cDC conventional dendritic cell, pDC plasmacytoid dendritic cell, MAST mast cell, Fib fibroblast, EPC epithelial cell, ENC endothelial cell

To identify potential interactions between the cell’s activation in the IC group, the expression patterns of the corresponding receptors and ligands (only chemokines, cytokines, and their receptors) were analyzed. The interaction pairs were mainly distributed on the surface of endothelial cells, macrophages, cDCs, and CD4^+^ Tem (Fig. 5a). The immune activation network based on the top50 interaction pairs in the IC group was presented in Fig. 5b. The pairs, such as TNF-TNFRSF1B, CD40-TNFSF13B, and TGF-β1-TGFβR1, were highly expressed across immune cells (Supplementary Fig. 4a).

In terms of overall cell–cell communication (Supplementary Fig. 3a), CCL15 was mainly expressed in bladder epithelial cells, whose receptors (CCR1, CCR3) were found in nearly all clusters. CX3CL1 was also mainly expressed in epithelial cells, whose receptor (CX3CR1) was upregulated in several downstream cell subsets. In addition, we found that two chemokine receptors (FGFR1 and FGFR2) were expressed across nearly all cell subpopulations, but their ligands (FGF2 and FGF10) were mainly expressed in the fibroblasts (Fig. 5c and Supplementary Fig. 4b). Moreover, the expression level of these ligands in the IC group was higher than that in the control group (Supplementary Fig. 4c). These findings implied that epithelial cells and fibroblasts might coordinate the transfer of immune cells in IC bladders. Nevertheless, it should be kept in mind that we included only CD45^+^ cells to perform downstream analysis, therefore, 5.58% of CD45^−^ cells were not representative (Fig. 1h). Further study is needed to verify these results. Regarding the immune cells, interaction pairs were mainly distributed on the surface of M2-like macrophages, inflammatory CD14^+^ macrophages, cDCs, and CD4^+^ Tem (Supplementary Fig. 3b), suggesting they were actively participating in the immunomodulation. The communications between these four clusters and other cell subsets are shown in Supplementary Fig. 3c. Based on special interaction pairs expressed on these cells (Supplementary Figs. 3d, e and 4d), the possible overall cell–cell communication network was constructed (Supplementary Fig. 3a).

The high expression level of inflammatory factors (TNF, IFNG, and IL-1β) indicated robust inflammatory responses in IC bladders (Fig. 5d and Supplementary Fig. 4d), which was accordant with the results of a previous study.^27^ In addition, CCL5, a newly discovered chemokine, was upregulated in nearly all cell types. The ELISA tests confirmed the high level of CCL5 in urine, but not in blood (Fig. 5e and Supplementary Fig. 4d), making it a potential urine biomarker for IC.

The decreased expression of IL-10 and IL-35 (Fig. 5f and Supplementary Fig. 4e) and the upregulated expression of TGF-β signaling pathway in Tregs were noted (Fig. 5g), suggesting the dysfunction of Tregs in IC bladders.^28^ Together with the CD8^+^ T exhaustion, the findings could possibly imply the dysfunction of immunomodulation in the IC group, which could lead to inflammatory continuation and fibrosis at the end.^29^ Several receptors (CCR7, CXCR4, IFNGR1/2, TGFβR2, and IL-2RG) were found upregulated in nearly all immune cells in the IC group (Supplementary Fig. 4f, g), indicating they could be served as potential therapeutic targets.

Apart from participating in immune responses, the DEGs of epithelial cells in IC patients were enriched in human papillomavirus infection and virus protein interaction with cytokine and cytokine receptor (Fig. 5h). Similar results were obtained in the enrichment analysis of fibroblasts (Fig. 5i). Subsequently, the top 5 transcription factors of epithelial cells in IC bladders were predicted, including NR2F1, FOXA1, HOXD1, TBX2, and OVOL1 (Fig. 5j). It is worth noticing that NR2F1 was associated with HIV infection.^30^ Moreover, FOXA1^31^ and TBX2^32^ were reported to be related to HPV infection. Regarding the cell types (Tregs, Th17, Act Bs, SMBs, USMBs, and neutrophils) with a large proportion change, we reviewed functional results of DEGs in these cells and found all of them were associated with virus infection (Fig. 5k). Taken together, we deduced that the virus infection might be one of the potential causes for IC. Thus, the urine samples from 20 IC patients and 14 controls were collected for virus detection. Notably, human polyomavirus-2 was detected with a positive rate of 95% (19/20) in the IC urine but not found in the urine of controls (Fig. 5l). This finding would provide novel clues and rationales for IC etiology and pathophysiological mechanisms, and more work and studies should be designed to deeply investigate cell types infected and their key receptors to find potential targets for the treatment of IC.

### ST demonstrated the aggregation of immune cells in IC urothelium

To generate unbiased transcriptomic maps of tissue sections, six bladder tissue samples from three patients with IC and three samples from the controls were mounted onto the spatially barcoded ST microarray slides (Fig. 1a and Table S3). After hematoxylin and eosin (H&E) staining and brightfield imaging, the slides were subjected to observing distinct histological features. Table S7 contained a summary of the data evaluation. After standard quality control, dimensionality reduction was performed using the Seurat methods, and visualization was realized through *t*-distributed Stochastic Neighbor Embedding (T-SNE). According to the DEGs in each cluster, stepwise cell clustering to construct a ST map was performed.

**Fig. 6:**
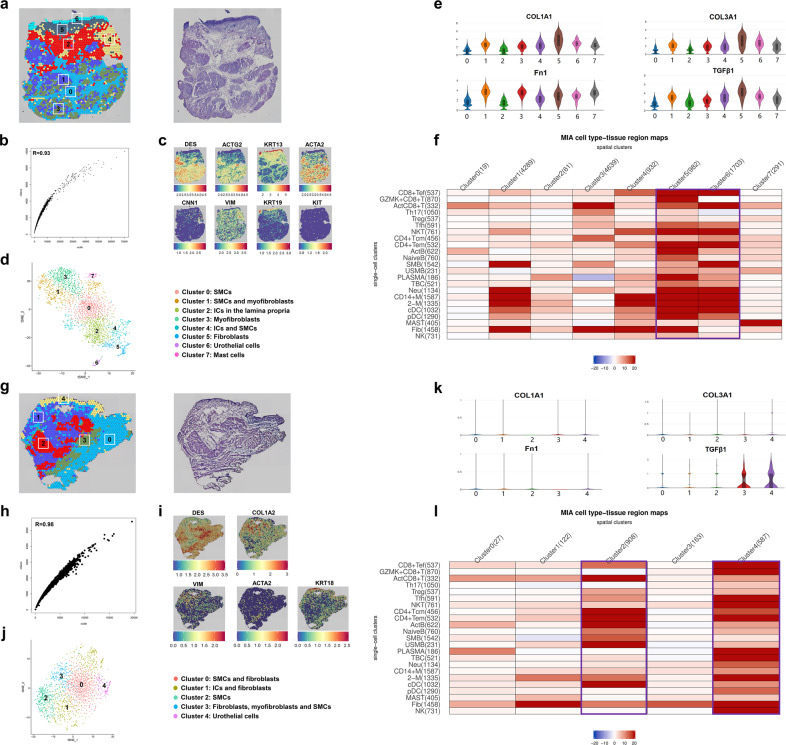
Mapping distinct immune populations across IC bladder sections using multimodal intersection analysis (MIA). **a**–**f** for the first IC bladder. **a** The spatial transcriptomics (ST) map of IC bladder (bar = 500 μm). **b** The correlation between the Genes and UMIs was calculated. **c** Spatial plots showing the marker genes for smooth muscle cells (SMCs), urothelial cells, myofibroblasts, interstitial cells (ICs), and mast cells. **d** T-SNE plot showing the clusters in the ST map of IC bladder. **e** Violin plots showing the expression of fibrotic genes (FN1, TGF-β1, COL1A1, and COL3A1) across the clusters. **f** Heatmap showing the distribution of immune cells in the ST map (the most mapped cell populations were labeled with purple box). **g**–**l** for control bladder, using the same presentation form with IC bladder. IC interstitial cystitis

Figure 6a presented the first ST map of IC bladder. A strong correlation between the Genes and UMIs was observed (Fig. 6b). Marker genes for smooth muscle cells (SMCs) (DES, ACTG2, and CNN1), urothelial cells (KRT19), myofibroblasts (ACTA2), interstitial cells (ISCs) (VIM and KRT13), and mast cells (KIT) were detected (Fig. 6c). The complete structure of the bladder wall including urothelial region, lamina propria, and muscle strata was depicted (Fig. 6d). Fibrotic genes, including FN1, TGF-β1, COL1A1, and COL3A1, were highly expressed across cell types, potentially indicating that the bladder structure could end up with severe fibrosis (Fig. 6e). Figure 6g showed the first ST map of the control bladder. A correlation coefficient of 0.98 was noted between the Genes and UMIs (Fig. 6h). SMCs, ISCs, fibroblasts (COL1A2), myofibroblasts, and urothelial cells were identified (Fig. 6i, j). However, a low expression level of fibrotic genes was noticed among the cells of the controls (Fig. 6k).

Based on the same procedures and protocols, another two ST views of IC bladders (Supplementary Figs. 5 and 7a–e) and two of control bladders (Supplementary Figs. 6 and 7g–k) were pictured. Interestingly, we noted that some immune cells aggregated in the urothelium in the ST map of IC, such as T cells in the second ST view (Supplementary Fig. 7a, b) and plasma cells in the third ST map (Supplementary Fig. 7d, e). However, this aggregation was not observed in the ST map of the control bladders (Supplementary Fig. 7g, h, j, k). The results further highlighted the important role of urothelium in the development of IC.

### Mapping of immune subpopulations revealed the distribution difference of immune cells in the IC and control bladder tissues

To pinpoint the location of immune cells in IC bladders, multimodal intersection analysis (MIA) was applied to interpret the spatially restricted mapping across the tissues. We spotted nearly all immune subpopulations such as macrophages, Tregs, resident memory CD8^+^ T cells, plasma cells, pDCs, cDCs, NKT cells, naive B cells, and neutrophils, were enriched in the urothelial region or located close to fibroblasts in IC bladders (Fig. 6f and Supplementary Fig. 7c, f), but they were discovered around urothelium and smooth muscle cells in control bladders (Fig. 6l and Supplementary Fig. 7i, l). Notably, only half of the immune cell subsets were identified in the urothelial region of control bladders. The results are highly consistent among the three samples in both IC and control bladders. The findings from MIA confirmed the important role of epithelial cells and fibroblasts in the development of IC, which were also suggested by cellphone DB analysis. To summarize, we successfully constructed an immune atlas on a spatial level and characterized an immune network in IC bladders.

## Discussion

To investigate the role of immunity in IC progress, we firstly sequenced CD45^+^ single cells and integrated scRNA-seq with ST through MIA to unravel the immune landscape of bladder of women with IC. A different distribution of immune cells was observed in the IC and control bladders, suggesting that urothelium may play a paramount role in the etiology and progress of IC. The damage to the urothelial stratum that can lead to its leakage and increased permeability is a crucial factor in bladder disease pathophysiology.^33^ In the present study, the multiple immune cell subtypes were activated by epithelial cells via chemokines CCL15-CCR1/3 and CX3CL1-CX3CR1. Various activated immune cells infiltrated into different layers of the bladder, including epithelial stratum, lamina propria, and muscle region located around fibroblasts. The activated immune cells triggered the dysfunction of cells in the bladder as well as the malformation of bladder structure, and then caused fibrosis, which eventually resulted in or deteriorated the IC-related lower urinary tract symptoms. Thus, it is important and pressing to find upstream causes of urothelial injury. Although this study evidenced that inflammation or immunity had an important role in IC progress, it seemed that it was more likely to be a downstream manifestation after the destruction of the epithelial barrier. In the future, more studies are needed to shed some light on this topic.

M2-like macrophages, inflammatory CD14^+^ macrophages, cDCs, and CD4^+^ Tem are the cells most involved in immunomodulation in IC bladders. Arazi et al.^34^ reported that M2-like macrophages could be served as an immunomodulatory center in lupus nephritis. Similarly, among inflammatory cells involved in rheumatoid arthritis (RA), macrophages and CD4^+^ Tems play a key role,^35,36^ which keeps in line with the current study. cDCs, homing by CCR6/CCL20 axis, involved in immunomodulation and were found increased in the synovial fluid in RA, idiopathic inflammatory myopathies dermatomyositis, and polymyositis.^37^ Taken together, these immune cells may underlie the key immunomodulation in the IC development and progress.

Currently, researchers have been focusing on the search of possible urinary and serum biomarkers for early detection of IC in patients. Previous studies showed that urinary inflammatory cytokines, including IL-2, IL-6, IL-8, IL-33, and IL-17A, were elevated in patients with IC, among which IL-33 was reported significantly increased in the urine of IC patients.^38^ There are other studies verifying chemokines as biomarkers for detection of IC in patients, for instance, a significant increase of CXCL10 in IC was noticed by Tyagi et al.^39^ Also, increased CXCR3 expression of infiltrating plasma cells in IC was reported.^40^ In this study, except for IL-1β, TNF, and IFNG, CCL5 was also found to be expressed on most immune cells, which was validated by its increased secretion in the urine of IC patients. CCL5 was upregulated in bladder tumor, urinary tract infection, reteropelvic junction obstruction, and IC.^41–44^ Since the tumor, infection, and information belonged to the content of exclusion criteria, CCL5 might be a potential biomarker for IC. Regarding the antibodies in serum, the increase of serum IgE in patients with IC was consistent with the findings in previous researches.^45^ Interestingly, a higher level of urine IgE was tested in patients with IC. Although the diagnosis of IC using cytokine urinary level as a marker has been established, a combination of several different urinary cytokines (e.g., CCL5 combined IgE in urine) as non-invasive markers might increase the accuracy of the results.

In conclusion, we constructed the immune landscape of the bladder in women with IC, and then confirmed the characteristics of these immune cell subsets and elaborated the relation and interaction within them. This study sets a precedent for investigating the immune atlas for IC. The immune landscape may provide profound insight into the pathophysiology of IC and work as the foundation for the diagnosis and treatment of this disease in the future.

## Materials and methods

### Study design and population

This prospective study (registration number: ChiCTR2000032176) was designed to investigate the immune atlas of the human bladder of female patients with IC. Human research was approved by the Medical Ethics Committee of West China Hospital, Sichuan University (#2019186). The study was performed after obtaining informed consent from all participants. The diagnosis of IC was in line with the National Institute of Diabetes, Digestive and Kidney Diseases guidelines.^1^ The characteristic pathological findings in the bladder wall were identified by cystoscopy (Fig. S1a). The inclusion criteria and exclusion criteria for IC patients were shown in Table S1. Female patients with pure stress urinary incontinence (SUI) but stable bladder function admitted for anti-incontinence surgery were offered enrolment as controls. The exclusion criteria of controls were presented in Table S2.

### Sample collection and processing

Urine and blood samples and bladder tissues were collected from patients and controls (Table S3). First, CD45^+^ single cells were isolated from bladder tissues to perform scRNA-seq (IC vs control group, *n* = 15 vs *n* = 9) and CyTOF (IC vs control group, *n* = 5 vs *n* = 5), respectively. Then, 6 bladder tissues from IC patients (*n* = 3) and controls (*n* = 3) were used for ST analysis. In addition, we performed hematoxylin and eosin stain (H&E) and immunofluorescence (IF) to demonstrate pathological features of IC or to validate specific cell subsets.

Once the samples were obtained during surgery, several pieces of bladder tissues were quickly frozen with liquid nitrogen and embedded with O.C.T. compound for H&E and IF assays. Bladder tissues with diameter of 1 cm were cut into small pieces (<1 mm in diameter) and digested with collagenase IV (1 mg/ml, Roche, Cat: 11088866001) and DNase I (0.1 mg/ml, Sigma-Aldrich, Cat: DN25) for 30 min on a 37 °C shaker. Subsequently, DMEM was added to dilute the suspension, and then a 40-μm cell container was used to filter the suspension. After centrifugation, the cell pellets were resuspended in 1 ml of ice-cold red blood cells lysis buffer following the manufacturer’s instruction. Finally, the pellet was resuspended in 5 ml PBS containing 0.04% weight/volume BSA. Ten microliters of suspension were counted under an inverted microscope with a hemocytometer. Trypan blue was used to quantify live cells, and the cell viability was over 80%. The single-cell suspensions prepared were used for scRNA-seq and CyTOF.

Besides, the bladder tissues (6.5 mm × 6.5 mm × 6.5 mm) from the participants were quickly frozen with isopentane and then embedded with O.C.T. compound for ST.

Peripheral venous blood samples and urine samples were obtained and stored in a −80 °C refrigerator before use.

### CD45^+^ single-cell preparation for scRNA-seq and CyTOF

Flow cytometry was applied to collected CD45^+^ single cells from samples with anti-CD45 antibody (Biolegend). Data analysis was performed with FlowJo (version 10). CD45^+^ single cells were subsequently used for scRNA-seq or CyTOF.

### Complementary DNA (cDNA) library construction for scRNA-seq

The cells that have passed the test were washed and resuspended to prepare a suitable cell concentration of 700–1200 cells/μl for 10× Genomics Chromium^TM^ (Single Cell 3’library and Gel Bead Kit v3). Gel Beads in Emulsion (GEMs) were constructed for single-cell separation according to the number of cells to be harvested. After GEMs were normally formed, they were collected for reverse transcription and barcode labeling. The GEMs were oil-treated, and the amplified cDNA was purified by magnetic beads and then subjected to cDNA amplification and quality inspection. The 3ʹ Gene Expression Library was constructed with the quality-qualified cDNA. After fragmentation, adaptor ligation, and sample index PCR, the library was finally quantitatively examined. The final library pool was sequenced in the Illumina Hiseq instrument using 150-base-pair paired-end reads.

### Quality control and clustering

Cell Ranger (version 5.0.0) was applied to process the raw data. A raw unique molecular identifier (UMI) count matrix was generated after Cell Ranger processing, then the matrix was converted into a Seurat object by R package Seurat.^46^ Cells with UMI numbers <1000, gene numbers <200 or >10,000, or with > 10% mitochondrial-derived UMI counts were considered low-quality cells and removed (Fig. 1c). To reduce the dimensionality of the scRNA-seq dataset, PCA was applied. The main cell clusters were identified with the FindClusters function offered by Seurat with resolution set as default (res = 0.9). They were then visualized with 2D UMAP. Conventional markers described in a previous study were used to categorize every cell into a known biological cell type,^47–49^ including B cells: CD79A; T cells: CD3E, CD4, and CD8A; myeloid cells: LYZ and CST3; fibroblasts: CLO1A1 and LUM; epithelial cells: KRT19 and UPK1B; NK cells: CD16/FCGR3A, CD56/NCAM1, and NKG7; and endothelial cells: CD31/PECMA1 (Fig. 1e-f). Subsequently, major cell types were further clustered into subclusters to detect heterogeneity within each cell type.

### Function analysis for differently expressed genes (DEGs)

The DEGs were calculated in the IC group in comparison with the control group. The false‐positive result was corrected by adjusted *p* value (adj. p value) using Benjamini–Hochberg method. The “adj. p value < 0.05” and “|logFC| > 0.5” were set as the cut‐off criterion. GO and KEGG enrichment analyses of significant DEGs were then performed. The false discovery rate <0.05 was set as the cut‐off criterion.

### Pseudotime analysis by Monocle2

Pseudotime analysis was conducted using Monocle2^50^ to determine the dramatic developmental trajectory and translational relationships. Significantly changed genes were identified by the differential GeneTest function in Monocle2 with a *q*-value <0.01.

### Cell–cell communication analysis with CellPhone DB

CellPhone DB 2 is a Python-based computational analysis tool, which enables the analysis of cell–cell communication at the molecular level.^51,52^ In the current study, immune cells were assessed using the software to determine interaction networks. Interaction pairs including chemokine/cytokine and their receptors were identified, of which *p* values <0.05 were returned by CellPhone DB, and selected for the evaluation of relationships between cell types.

### Mass cytometry (CyTOF)

A panel of 42 metal isotope-tagged antibodies (Table S6) was used to evaluate the immune cell populations in the human bladder tissue. Briefly, fresh CD45^+^ single cells were washed and incubated with cisplatin cocktail for viability, and then was fixed in 1.6% formaldehyde and permeabilized. Cells were then stained with the antibody cocktail and incubated with intercalation solution followed by mixing with EQ Four Element Calibration Beads and harvesting with a Helios mass cytometer.

The data were acquired using a Helios system (Fluidigm Sciences) at ≤500 events per second. All CyTOF files were normalized and manually gated in FlowJo (version 10). The data were subjected to the metal isotope beads normalization method. The CD45 gated cell populations were clustered using the X-shift algorithm that was performed on pooled samples to automatically identify underlying immune subsets.^53^ Normalized marker expression levels were visualized as heatmaps. Cell frequency in each cluster was calculated as the assigned cell events dividing the total cell events in the same sample after manual gating. T-SNE dimension reduction was performed using R package. The cell types were distinguished by canonical markers.

### Sample preparation for ST

The bladder tissues (6.5 mm × 6.5 mm × 6.5 mm) from all participants were snap-frozen with precooled isopentane and then embedded with O.C.T. compound. Cryosections were cut with 10 μm thickness and mounted onto the GEX arrays. Sections were placed on Thermocycler Adaptor with the active surface facing up and incubated 1 min at 37 °C, and then were fixed for 30 min with methyl alcohol in −20 °C followed by staining with H&E (Eosin, Dako CS701, Hematoxylin Dako S3309, bluing buffer CS702).^54^ The brightfield images were taken via a Leica DMI8 whole-slide scanner at ×10 resolution.

### Permeabilization and reverse transcription

Visum spatial gene expression was processed using Visum spatial gene expression slide and Reagent Kit (10× Genomics, PN-1000184). For each well, Slide Cassette was used to create leakproof wells for adding reagents. In all, 70 μl permeabilization enzyme was added and incubated at 37 °C for 20 min. Each well was washed with 100 μl SSC, and 75 μl reverse transcription Master Mix was added for cDNA Synthesis.

### cDNA library preparation

At the end of first-strand synthesis, RT Master Mix was removed from the wells with the subsequent addition of 75 μl 0.08 M KOH and incubated 5 min at room temperature. KOH was then removed from wells, and the residuals were washed with 100 μl EB buffer. In all, 75 μl Second Strand Mix was added to each well for second-strand synthesis. cDNA amplification was performed on a S1000TM Touch Thermal Cycler (Bio-Rad).

According to the manufacturer’s introduction, Visum spatial libraries were constructed using Visum spatial Library construction kit (10× Genomics, PN-1000184). The libraries were finally sequenced using an Illumina Novaseq6000 sequencer with a sequencing depth of at least 100,000 reads per spot with a pair-end 150 bp (PE150) reading strategy (CapitalBio Technology, Beijing).

### Quality control and clustering

Generally, alignment, filtering, barcode counting, and UMI counting were performed with Spaceranger (version 1.1.0) count module to generate feature-barcode matrix and determine clusters (Table S7). Dimensionality reduction was performed using PCA, and visualization was realized by T-SNE. The resolution of cluster is 0.8. The canonical marker genes^47–49^ were used to identify different cell subsets in ST, including DES, CNN1, TAGLN, ACTG2, MYL9, MYH11, and TPM2 for smooth muscle cells (SMCs); LUM, FBLN1, COL1A1, COL1A2, and COL3A1 for fibroblasts; MYLK, THY1 and ACTA2 for myofibroblasts; VIM and KRT13 for intermediate cells (ISCs); and KRT17, KRT18, KRT19 and UPK1A for urothelial cells.

### Construction of a structural immune atlas via multimodal intersection analysis (MIA)

MIA is an analytical method that integrates single-cell expression profiles with spatial transcriptome data.^21^ This analysis is proceeded by first delineating sets of cell type-specific and tissue region-specific genes and then determining whether their overlap is higher (enrichment) or lower (depletion) than expected by chance. After obtaining two kinds of gene sets, the degree of significant enrichment was evaluated by co-expression genes and hypergeometric test.

### H&E and IF

In all, 4-μm frozen sections were developed for H&E and IF using bladder tissues embedded in the O.C.T. compound. IF was performed to validate specific clusters with following primary antibodies: anti-CD68 (mouse, 2 µg/ml, Abcam, ab201340); anti-S100A8 (rabbit, 1:200, Bioss, bs-2696R) and CD163 (rabbit, 1:200, Bioss, bs-2527R). Fluorescently conjugated secondary antibodies included Rhodamine (TRITC) AffiniPure Goat Anti-Mouse IgG (red) (ZENBIO, 511102) and Goat Anti-Rabbit IgG (H&L)–Alexa Fluor 488 (green) (ZENBIO, 550037). The procedures were conducted based on the manufacturer’s recommendations.

### ELISA

CCL5 (CCL5 Human ELISA Kit, RD, DRN00B) was measured by ELISA in plasma and urine collected from IC patients and controls according to the manufacturer’s recommendations.

### Detection of immunoglobulin

IgA, IgM, IgG, and IgE from plasma and urine of patients and controls were detected using the immunoturbidimetry method by au680 biochemical analyzer (Beckman Coulter, Inc.). IgE in the urine was tested using the ELISA kit (BioVision, E4471-100).

### Detection of virus

High-throughput sequencing technology was applied to analyze the nucleic acid sequence of microorganisms of urine from 20 patients with IC and 14 controls (Table S3), then the microorganisms were identified by comparing with existed results in the database.

### Statistical analysis

All statistical analyses and graph generations were performed in R (version 3.6.1) and GraphPad Prism software (version 8.0). Continuous variables are presented as the means ± standard deviation, and differences between groups were tested using the Mann–Whitney *U*-test, two-tailed. The difference of cell frequency in each cluster was calculated by chi-square test. A *p* value < 0.05 was considered significant.

## Supplementary information

Supplementary Materials (Figure and Table)

Supplementary Fig. 1

Supplementary Fig. 2

Supplementary Fig. 3

Supplementary Fig. 4

Supplementary Fig. 5

Supplementary Fig. 6

Supplementary Fig. 7

## Data Availability

The data used for the current study are available from the corresponding author upon request (luodeyi1985@163.com). Experimental protocols and the data analysis pipeline used in our work follow the 10× Genomics and Seurat official websites. There are no custom codes for data analysis in the current study.
