# Supplementary material for: Integrating single-cell RNA sequencing with spatial transcriptomics reveals immune landscape for interstitial cystitis
Source: Signal Transduct Target Ther. 2022 May 20;7:161. doi: 10.1038/s41392-022-00962-8 (PMC9120182; doi:10.1038/s41392-022-00962-8)
Supplement: Supplementary file 1 — Supplementary Materials (Figure and Table) [file 41392_2022_962_MOESM1_ESM.pdf]

Supplementary Materials for

Integrating single-cell RNA sequencing with spatial transcriptomics reveals immune landscape for interstitial cystitis

Liao Peng[1, †], Xi Jin[1, †], Bo-ya Li[1], Xiao Zeng[1], Bang-hua Liao[1], Tao Jin[1], Jia-wei Chen[1], Xiao-shuai Gao[1], Wei Wang[1], Qing He[1], Guo Chen[1], Li-Na Gong[1], Hong Shen[1], Kun-jie Wang[1], Hong Li[1, *], De-yi Luo[1, *]

Correspondence to: luodeyi1985@163.com and lihonghxhx@scu.edu.cn

This PDF file includes:

Supplementary Figure S1 to S7

Supplementary Table S1 to S7

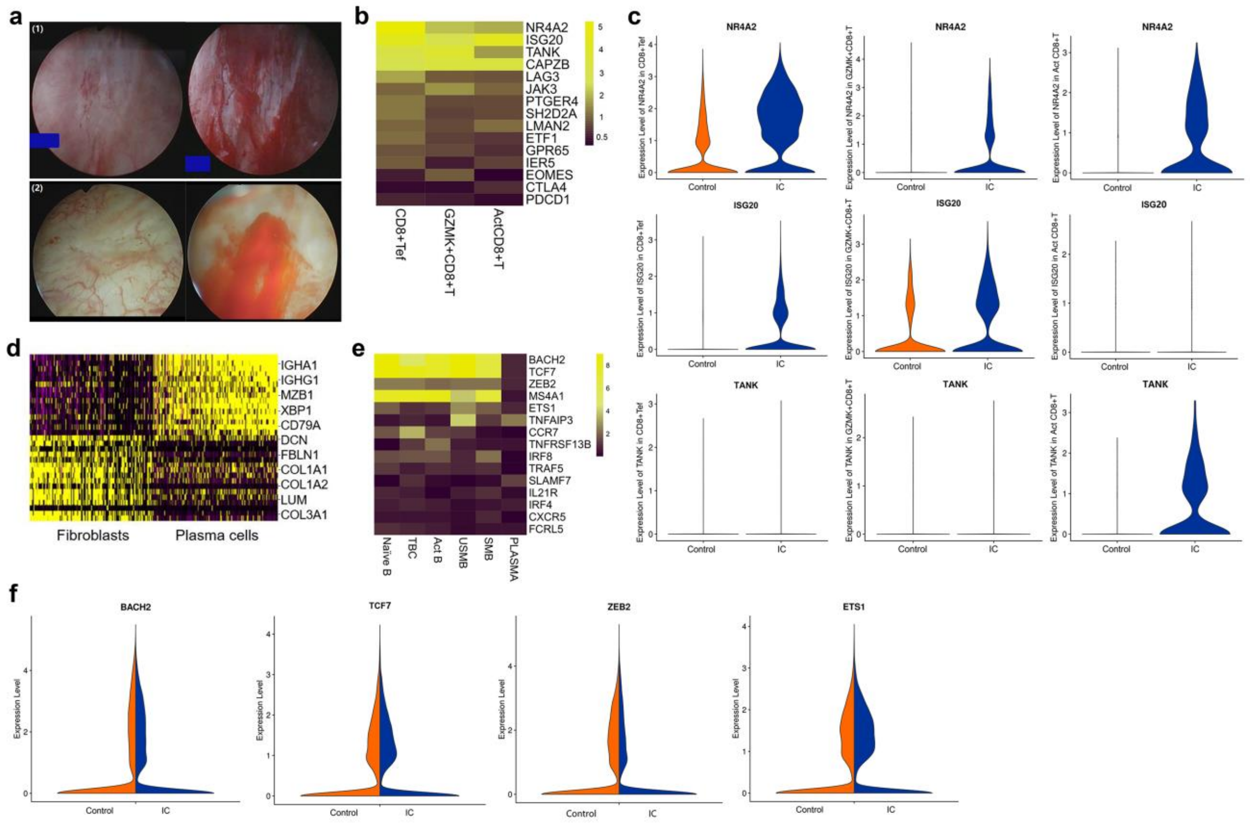

Supplementary Fig. 1 The expression level of selected genes in T-cell lineages and B-cell subsets. a Characteristic pathological findings of IC were identified by cystoscopy. **b** The expression of exhausted genes three $CD8^+$ T-cell clusters. **c** The expression levels of representative exhausted markers in each cell type in the two groups. **d** The expression of marker genes of plasma cells and fibroblasts. **e** The expression of age-related genes in B cells. **f** The expression level of representative age-related genes in the two groups. IC, interstitial cystitis; $CD8^+$ Tef, $CD8^+$ effector T cell; $CD4^+$ Tcm, central memory $CD4^+$ T cell; Treg, regulatory T cell; $CD4^+$ Tem, effector memory $CD4^+$ T cell; Tfh, follicular helper T cell; Act $CD8^+$ T, activated $CD8^+$ T cell; NK, $CD56^{bright}$ $CD16^{low}$ natural killer cell; Act B, activated B cell; SMB, switched memory B cell; USMB, un-switched memory B; PLASMA, plasma cell; TBC, transitional B cell.

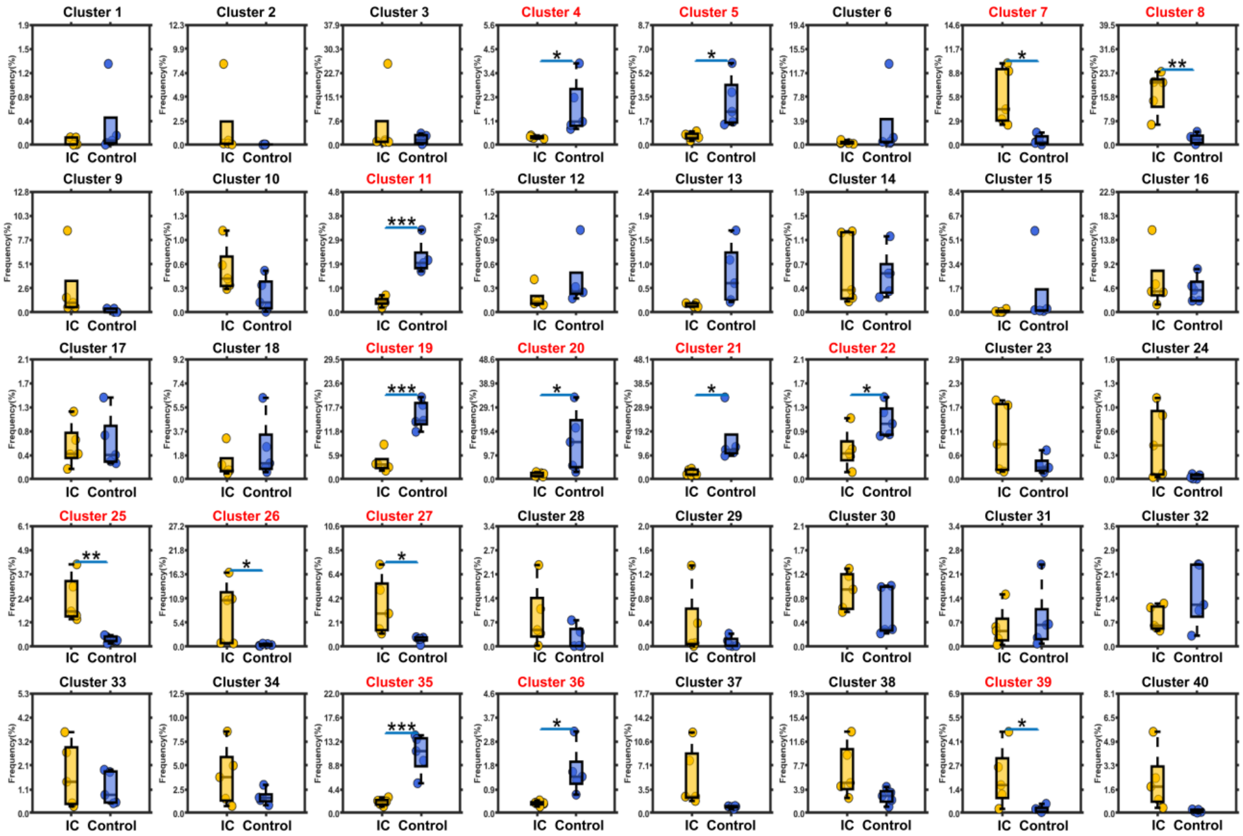

Supplementary Fig. 2 The cell frequency of each cluster revealed by mass cytometry (Mann-Whitney U-test, two-tailed, *p＜0.05; **p＜0.01; ***p＜0.001, IC, n=5; control, n=5). IC, interstitial cystitis.

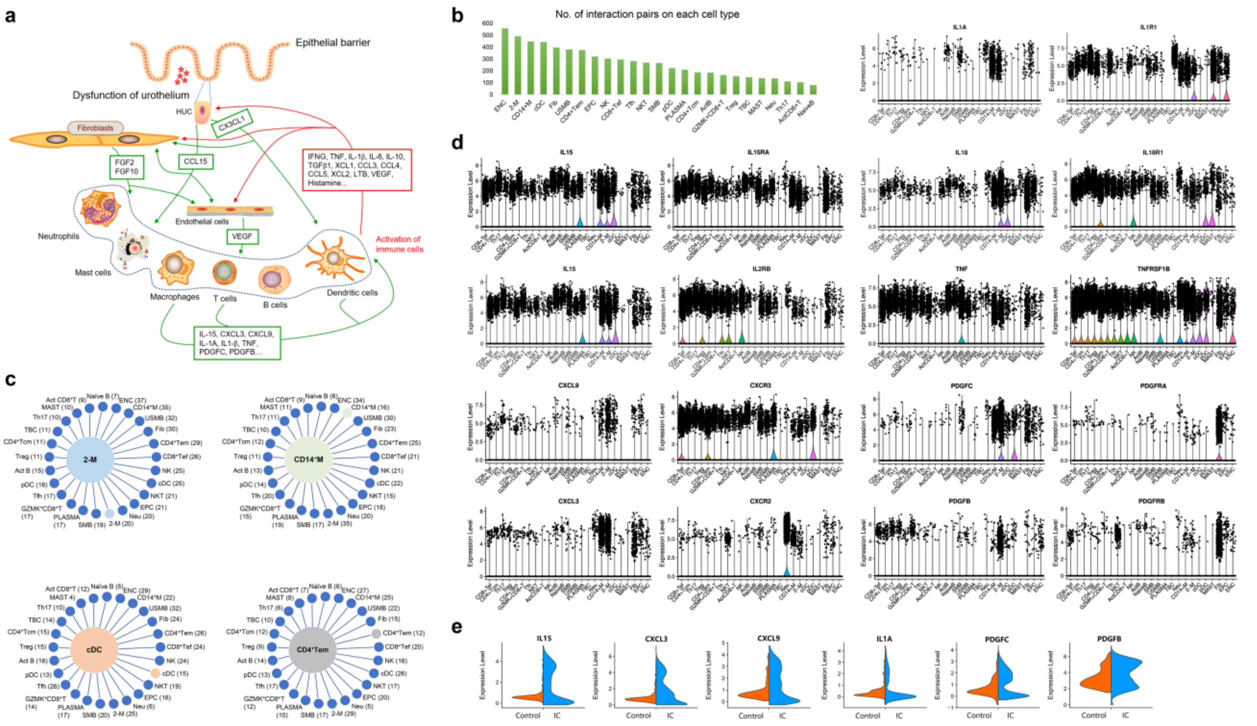

Supplementary Fig. 3 The overall cell-cell communication network in the IC bladders. a The communication network in the IC bladders. **b** The distribution of interaction pairs across all cell types. **c** The communications between the M2-like macrophages, inflammatory CD14$^+$ macrophages, cDCs, and CD4$^+$ Tem and other cell subsets. **d** The representative interaction pairs to link M2-like macrophages, inflammatory CD14$^+$ macrophages, cDCs, and CD4$^+$ Tem and other cell types. **e** The expression level of representative ligands in the two groups. IC, interstitial cystitis.

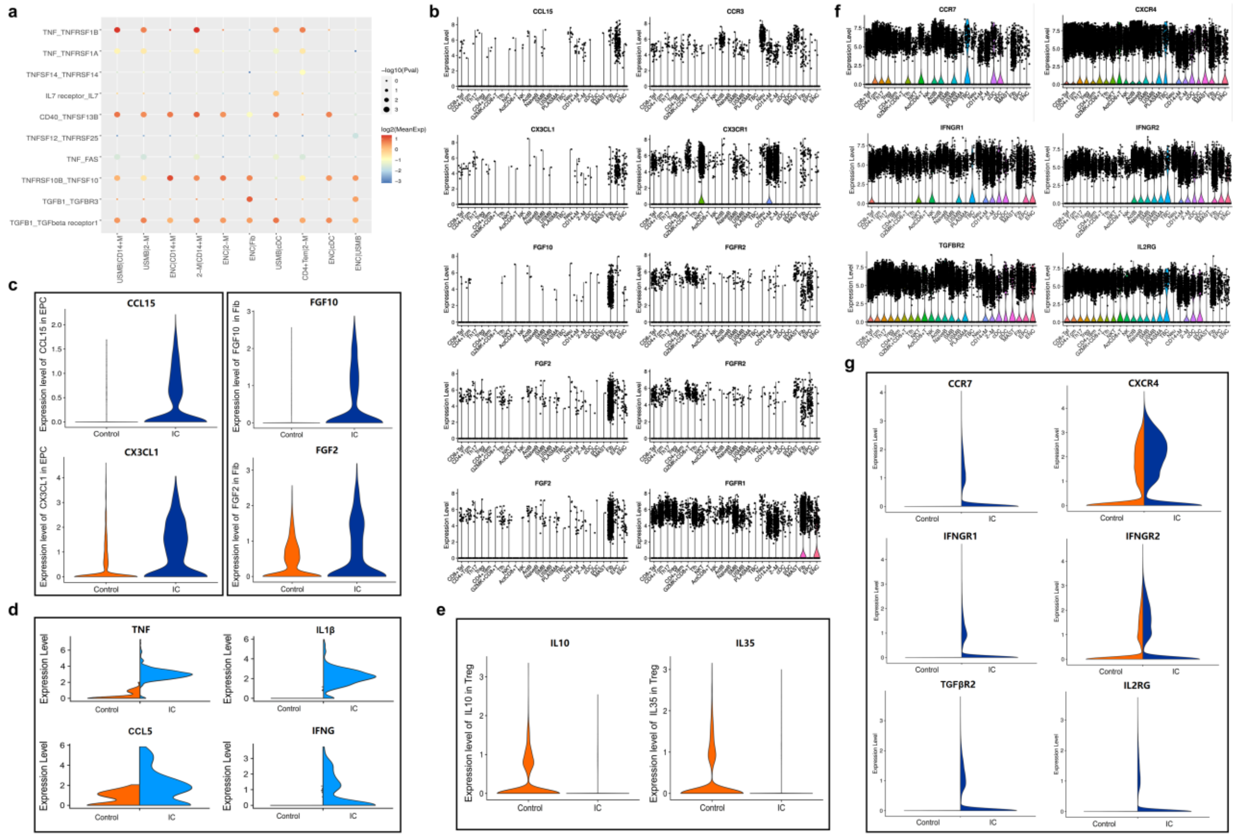

Supplementary Fig. 4 Cellphone DB analysis revealed important interaction pairs (IPs) across clusters. a Top 10 IPs across clusters. **b** Violin plots showing the IPs expressed specifically on epithelial cells and fibroblasts. **c** The expression level of representative ligands in the two groups. **d** The expression level of selected inflammatory genes in the two groups. **e** The expression level of IL-10 and IL-35 in the two groups. **f-g** The representative upregulated receptors in IC bladders. IC, interstitial cystitis; CD8$^+$ Tef, CD8$^+$ effector T cell; CD4$^+$ Tcm, central memory CD4$^+$ T cell; Treg, regulatory T cell; CD4$^+$ Tem, effector memory CD4$^+$ T cell; Tfh, follicular helper T cell; Act CD8$^+$ T, activated CD8$^+$ T cell; NK, CD56^{bright} CD16^{low} natural killer cell; Act B, activated B cell; SMB, switched memory B cell; USMB, un-switched memory B; PLASMA, plasma cell; TBC, transitional B cell; Neu, neutrophil; CD14$^+$ M, inflammatory CD14$^+$ macrophage; 2-M, M2-like macrophage; cDC, conventional dendritic cell; pDC, plasmacytoid dendritic cell; MAST, mast cell; Fib, fibroblast; EPC, epithelial cell; ENC, endothelial cell.

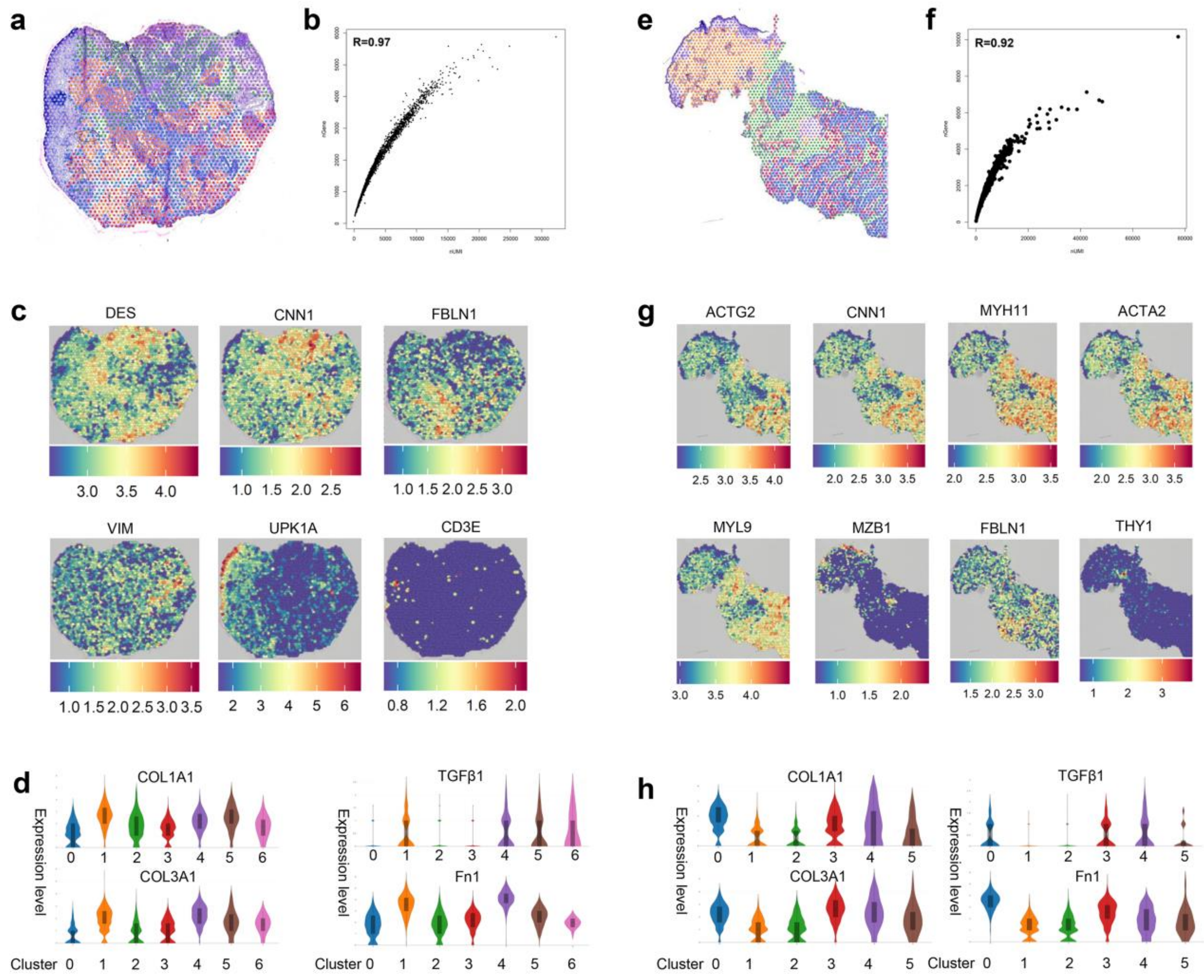

Supplementary Fig. 5 Spatial profiles of two IC bladders. a-d for the second IC bladder. **a** The spatial transcriptomic (ST) map of IC bladder. **b** The correlation between the Genes and UMIs was calculated. **c** Spatial plots showing the expression of marker genes for smooth muscle cells, urothelial cells, fibroblasts, myofibroblasts, and interstitial cells. **d** Violin plots showing the expression level of fibrotic genes. **e-h** for the third IC bladder, using the same presentation form with the second IC bladder. IC, interstitial cystitis.

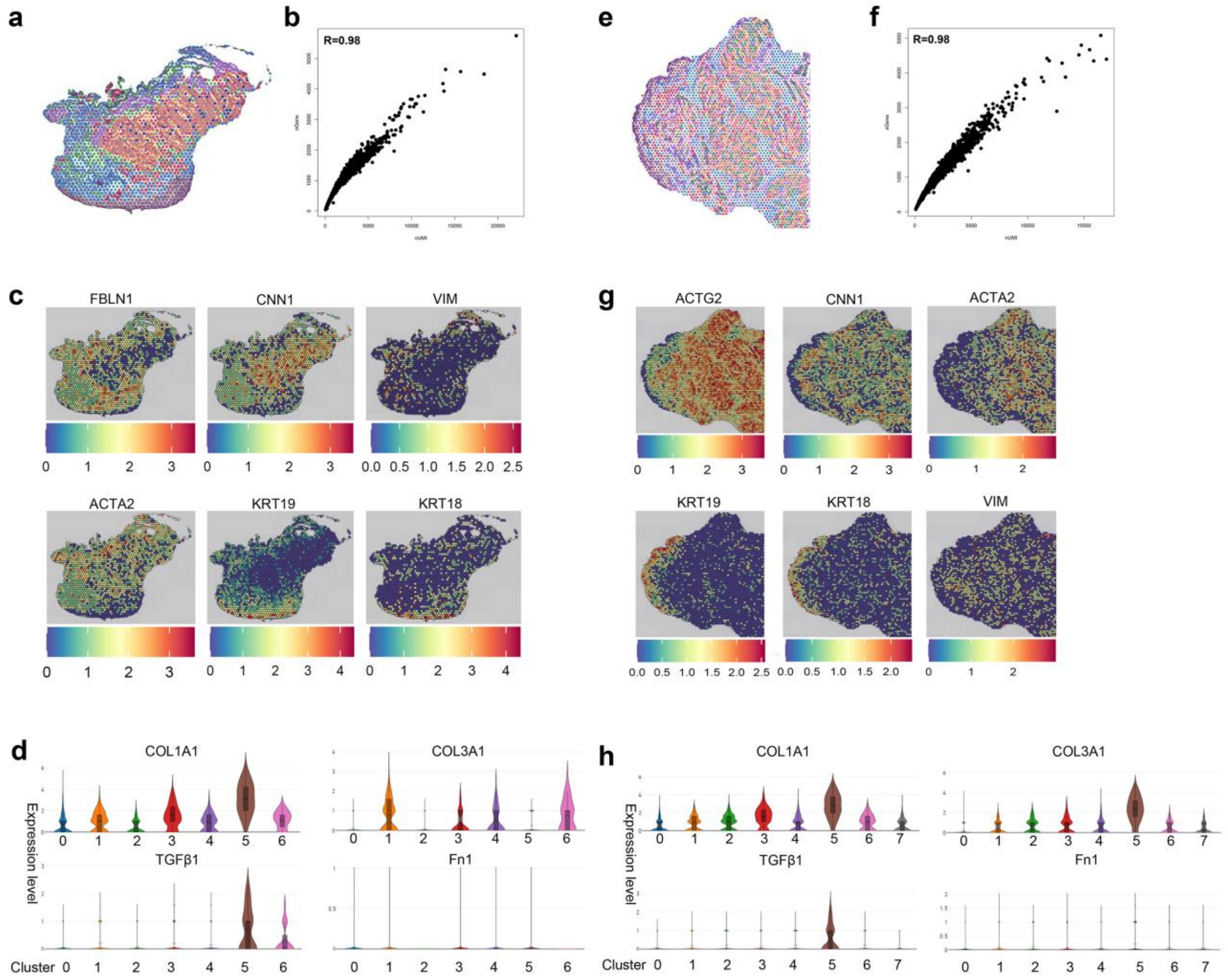

Supplementary Fig. 6 Spatial profiles of two control bladders. a-d for the second control bladder. **a** The spatial transcriptomic (ST) map of control bladder. **b** The correlation between the Genes and UMIs was calculated. **c** Spatial plots showing the expression of marker genes for smooth muscle cells (SMCs), urothelial cells, fibroblasts, myofibroblasts, and interstitial cells. **d** Violin plots showing the expression level of fibrotic genes. **e-h** for the third control bladder, using the same presentation form with the second control bladder.

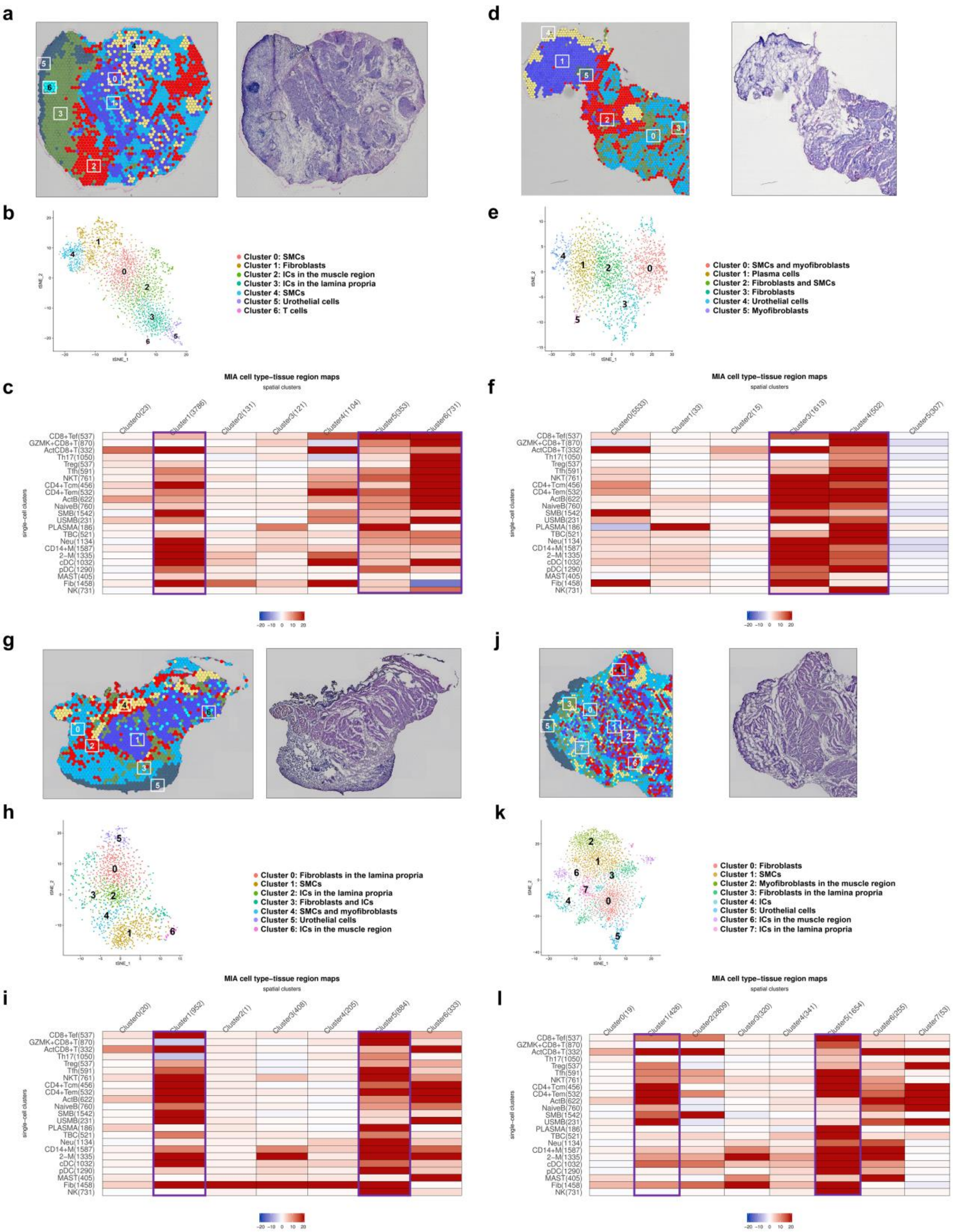

Supplementary Fig. 7 Mapping of immune subpopulations using multimodal intersection analysis (MIA). a-c for the second IC bladder. **a** The spatial transcriptomic (ST) map of IC bladder (bar=500um). **b** T-SNE plot showing the clusters in the ST map of IC bladder. **c** Heatmap showing

the distribution of immune cells in the ST map (the most mapped cell populations were labeled with purple box). **d-f** for the third IC bladder. **g-i** for the second control bladder. **j-l** for the third control bladder. IC, interstitial cystitis; ICs, interstitial cells; SMC, smooth muscle cells.

Table S1 Inclusion and exclusion criteria of the patients with interstitial cystitis.

Inclusion criteria

1) Patients over 18 years old;
2) Previously diagnosed with interstitial cystitis for a duration of >6 months;
3) The characteristic pathological findings in the bladder wall were identified by cystoscopy;
4) O'Leary-Sant Interstitial Cystitis Symptom and Problem Index score over 18;
5) Patients understand the purpose of this study as explained by the investigator and that they can withdraw from the study at any time.

Exclusion criteria

1. General conditions

1) The investigators assessed that she was not suitable for the study;
2) Currently diagnosed with cancer, or have previous history of cancer within the preceding 5 years;
3) Currently diagnosed with severe heart, lung, liver, kidney, or blood disorder;
4) Patients who are pregnant, pregnant women or lactating women or women who desire to become pregnant.

2. Urological problems

1) Have previous history of urinary infection (e.g., bacterial cystitis, bladder tuberculosis, urethritis, genital chlamydia infection, and genital herpes) within 12 weeks;
2) Currently diagnosed with any of following diseases, and/or current urinary symptoms (i.e., bladder pain, bladder discomfort, urinary frequency, persistent urge to urinate, and/or urinary urgency) are caused primarily by these diseases:
    a. Bladder diseases (overactive bladder, neurogenic bladder, bladder stone, radiation cystitis)
    b. Urethral diseases (urethral diverticulum, urethral stricture, urethral stone)
    c. Gynaecological diseases (endometriosis, uterine fibroids, vaginitis, menopausal syndrome, pelvic organ prolapse)
    d. Others (neurogenic urinary frequency, polyuria)
3) Have previous history of augmentation cystoplasty or cystectomy;
4) Have previous history of chemical compound (such as cyclophosphamide) derived cystitis.

3. Treatment related

1) Have history of the following therapies within 24 weeks: Hydrodistension, intravesical laser therapy, intravesical electrical coagulation, transurethral resection, pelvic reconstructive surgery, nerve block or spinal cord stimulation for pain relief;
2) Received intravesical instillation of any drugs within 12 weeks.

Table S2 Exclusion criteria of the controls.

(1) with urologic cancers, stones, mixed urinary incontinence, infection, or congenital malformation of urinary system;
(2) with liver and thyroid diseases;
(3) with a history of radiotherapy and chemotherapy;
(4) with distinct immune systematic disorders such as systemic lupus erythematosus, Sjogren's syndrome, and autoimmune hepatitis;
(5) she declined to participate or could not preform the study.

Table S3 The baseline information of the included patients with interstitial cystitis (IC) and controls with SUI.

	Diagnosis	Gender	Age (years)	Duration (months)	Menopause	Height (cm)	Weight (kg)	BMI	scRNA-seq	ST	H&E	IF	CyTOF	ELISA	Virus detection
IC 1	IC	Female	78	120	Post menopause	150	63	28.00	√		√	√		√	√
IC 2	IC	Female	72	96	Post menopause	148	50	22.83	√		√	√		√	√
IC 3	IC	Female	80	84	Post menopause	150	40	17.78	√		√	√		√	√
IC 4	IC	Female	65	80	Post menopause	158	75	30.04	√		√	√		√	√
IC 5	IC	Female	70	56	Post menopause	156	66	27.12	√	√	√	√		√	√
IC 6	IC	Female	68	34	Post menopause	155	59	24.56	√		√	√		√	√
IC 7	IC	Female	62	20	Post menopause	155	56	23.31	√		√	√		√	√
IC 8	IC	Female	66	24	Post menopause	157	57	23.12	√		√	√		√	√
IC 9	IC	Female	74	65	Post menopause	158	51	20.43	√		√	√		√	√
IC 10	IC	Female	72	60	Post menopause	157	57	23.12	√	√	√	√		√	√
IC 11	IC	Female	61	24	Post menopause	150	49	21.78	√		√	√		√	√
IC 12	IC	Female	63	47	Post menopause	150	55	24.44	√		√	√		√	√
IC 13	IC	Female	69	75	Post menopause	156	57	23.42	√	√	√	√		√	√
IC 14	IC	Female	79	60	Post menopause	168	60	21.26	√		√	√		√	√
IC 15	IC	Female	49	40	Premenopausal	160	42	16.41	√		√	√		√	√
IC 16	IC	Female	56	36	Post menopause	158	62	24.84					√	√	√
IC 17	IC	Female	63	47	Post menopause	168	73	25.86					√	√	√
IC 18	IC	Female	67	28	Post menopause	158	52.5	21.03					√	√	√
IC 19	IC	Female	50	36	Post menopause	154	49.5	20.87					√	√	√
IC 20	IC	Female	58	37	Post menopause	163	60	22.58					√	√	√
SUI 1	SUI	Female	70	-	Post menopause	158	64	25.64	√	√	√	√		√	√
SUI 2	SUI	Female	76	-	Post menopause	168	55	19.49	√		√	√		√	√
SUI 3	SUI	Female	62	-	Post menopause	165	54	19.83	√		√	√		√	√
SUI 4	SUI	Female	66	-	Post menopause	158	60	24.03	√		√	√		√	√
SUI 5	SUI	Female	60	-	Post menopause	160	52	20.31	√		√	√		√	√
SUI 6	SUI	Female	67	-	Post menopause	164	68	25.28	√	√	√	√		√	√
SUI 7	SUI	Female	60	-	Post menopause	156	54	22.19	√		√	√		√	√
SUI 8	SUI	Female	68	-	Post menopause	158	64	25.64	√	√	√	√		√	√
SUI 9	SUI	Female	76	-	Post menopause	162	49	18.67	√		√	√		√	√
SUI 10	SUI	Female	72	-	Post menopause	150	45	20.00					√	√	√
SUI 11	SUI	Female	66	-	Post menopause	165	57	20.94					√	√	√
SUI 12	SUI	Female	65	-	Post menopause	149	54	24.32					√	√	√
SUI 13	SUI	Female	66	-	Post menopause	170	65	22.49					√	√	√
SUI 14	SUI	Female	67	-	Post menopause	160	65	25.39					√	√	√

SUI, stress urinary incontinence; scRNA-seq, single cell RNA sequence; ST, spatial transcriptomics; H&E, immunohistochemistry; IF, immunofluorescence; CyTOF, mass cytometry; ELISA, enzyme linked immunosorbent assay; BMI, body mass index; ICSI, O'Leary-Sant interstitial cystitis symptom index; ICPI, O'Leary-Sant interstitial cystitis problem index; VAS, visual analog scale; BOTX-A, botulinum toxin type A injection; 1, yes; 0, no

Continued table S3 The baseline information of the included patients with interstitial cystitis (IC) and controls.

	Voids in 24h	No. of leaks*	Nocturia episodes	O'Leary Sant ICSI	O'Leary Sant ICPI	VAS (0-10)	History of oral medication	Previous hydrodistension	Previous BOTX-A injection	Hypertension	Diabetes mellitus
IC 1	30	-	10	19	14	8	1	1	1	0	1
IC 2	41	-	6	16	16	7	1	1	0	0	1
IC 3	36	-	14	19	13	8	1	1	1	1	1
IC 4	20	-	16	16	15	9	1	1	0	1	0
IC 5	36	-	8	17	13	6	1	1	0	0	1
IC 6	29	-	6	18	16	7	1	1	0	1	1
IC 7	24	-	9	17	15	5	1	1	1	1	0
IC 8	19	-	6	18	16	6	1	1	0	0	1
IC 9	27	-	2	14	13	3	1	1	1	1	0
IC 10	20	-	1	17	11	4	1	1	0	0	0
IC 11	14	-	3	13	13	7	1	1	0	0	1
IC 12	34	-	2	16	12	5	1	1	0	1	0
IC 13	17	-	1	15	15	6	1	1	0	1	0
IC 14	18	-	3	12	16	9	1	1	1	0	0
IC 15	20	-	3	15	14	6	1	1	0	0	0
IC 16	16	-	4	19	14	5	1	1	1	0	1
IC 17	18	-	5	15	13	9	1	1	0	0	0
IC 18	19	-	6	18	13	8	1	1	0	1	0
IC 19	18	-	4	17	11	6	1	1	1	0	0
IC 20	21	-	3	16	10	8	1	1	1	0	0
SUI 1	6	2	0	2	3	0	1	0	0	0	1
SUI 2	6	3	1	5	3	0	0	0	0	0	0
SUI 3	7	3	1	4	2	0	0	0	0	0	1
SUI 4	5	3	0	3	1	0	0	0	0	1	0
SUI 5	5	2	0	4	2	0	0	0	0	1	0
SUI 6	6	3	0	2	4	1	1	0	0	0	0
SUI 7	6	3	0	4	2	0	0	0	0	0	0
SUI 8	6	2	1	2	5	1	0	0	0	0	0
SUI 9	7	4	0	6	3	0	0	0	0	0	0
SUI 10	6	3	0	2	4	0	0	0	0	0	1
SUI 11	7	3	0	2	2	0	0	0	0	0	0
SUI 12	6	2	0	0	0	0	0	0	0	0	0
SUI 13	5	2	0	0	0	0	0	0	0	0	0
SUI 14	6	1	0	0	0	0	0	0	0	0	0

SUI, stress urinary incontinence; scRNA-seq, single cell RNA sequence; ST, spatial transcriptomics; H&E, immunohistochemistry; IF, immunofluorescence; CyTOF, mass cytometry; ELISA, enzyme linked immunosorbent assay; BMI, body mass index; ICSI, O'Leary-Sant interstitial cystitis symptom index; ICPI, O'Leary-Sant interstitial cystitis problem index; VAS, visual analog scale; BOTX-A, botulinum toxin type A injection; 1, yes; 0, no

* The number of leaks was counted when a SUI patient had an involuntary urine leakage with an increased abdominal pressure such as coughing in their daily life

Table S4 Basic information for single-cell RNA sequencing.

Estimated Number of Cells	Fraction Reads in Cells	Median Genes per Cell	Median UMI Counts per Cell	Chemistry	Transcriptome	Pipeline Version
135,091	0.846	1,613	3,979	Single Cell 3' v3	GRCh38	Cellranger-5.0.0

Table S5 Top 100 genes of natural killer cells.

Gene	Avg_log2 FC	Pct.1	Pct.2	P_val_adj
GNLY	3.566755424	0.569	0.063	0
XCL2	2.742127035	0.738	0.109	0
XCL1	2.627482664	0.664	0.058	0
IL12RB2	2.261224206	0.584	0.164	5.4E-272
PDE7B	1.971469766	0.35	0.099	4E-143
TRDC	1.950972857	0.601	0.029	0
KLRD1	1.941195695	0.713	0.131	0
CD7	1.932342935	0.911	0.405	0
KLRC1	1.857884176	0.546	0.02	0
KLRF1	1.833426333	0.525	0.028	0
CTSW	1.784223163	0.693	0.161	0
NCALD	1.767861624	0.688	0.135	0
AREG	1.706684855	0.711	0.296	7.1E-208
KLRB1	1.699612513	0.787	0.213	0
GRAMD1B	1.699283959	0.492	0.185	1.5E-142
ATP8B4	1.652476407	0.612	0.077	0
NCAM1	1.633024822	0.481	0.036	0
METRNL	1.578152008	0.681	0.258	2.2E-233
TXK	1.541200018	0.611	0.159	0
NKG7	1.469668185	0.734	0.284	8.8E-218
LINC00299	1.459365733	0.432	0.07	0
TNFRSF18	1.451057441	0.603	0.095	0
TIAM1	1.443543758	0.495	0.193	1.9E-136
AHI1	1.420023617	0.655	0.318	6.9E-145
KIT	1.391382173	0.272	0.009	0
REL	1.367390103	0.85	0.682	1.1E-103
SAMD3	1.343457709	0.597	0.197	3.2E-206
IL2RB	1.338806557	0.649	0.189	0
MAP3K8	1.334258947	0.756	0.376	2E-174
MCTP2	1.326572631	0.654	0.274	2E-166
RIN3	1.324658895	0.748	0.239	0
NFKB1	1.306638235	0.831	0.573	9E-125
GNG4	1.277933889	0.262	0.047	3E-188
SYTL3	1.266067822	0.859	0.582	1.6E-126
SSBP2	1.255973249	0.596	0.363	1.26E-64
STAT4	1.255705081	0.891	0.544	1.4E-152
PLCB1	1.251880005	0.736	0.236	3.5E-256
CD247	1.235241644	0.757	0.497	5.1E-99
CLIC3	1.229989095	0.461	0.095	1.4E-295
NCR1	1.228202655	0.469	0.023	0
LDB2	1.225377444	0.299	0.032	0
CMC1	1.221800688	0.586	0.29	1.9E-113
GZMB	1.219984372	0.435	0.162	4.11E-97
ABTB2	1.211089342	0.252	0.102	2.19E-46
AGPAT4	1.205883055	0.48	0.151	1.1E-171
FAM177A1	1.203877145	0.729	0.508	4.45E-84
CNOT2	1.202664034	0.712	0.475	3.46E-85
ZBTB16	1.172210902	0.431	0.082	2E-301
RASSF8	1.161245528	0.301	0.024	0
PDE4A	1.152454345	0.594	0.203	2E-205
ATP10A	1.1391751	0.405	0.129	1.6E-133
MATK	1.127499229	0.564	0.108	0
AOAH	1.116482658	0.696	0.399	3.95E-89

SH2D1B	1.11366249	0.368	0.007	0
LINGO2	1.109284041	0.17	0.01	0
PCDH9	1.101449026	0.184	0.039	4.1E-103
KCNQ5	1.098838719	0.483	0.166	2.9E-134
PIK3R1	1.097248419	0.769	0.491	7.7E-99
FOSL2	1.097052818	0.678	0.33	1.3E-146
KRT86	1.096020982	0.324	0.005	0
YES1	1.079713337	0.583	0.194	6.4E-188
B3GNT7	1.060041236	0.394	0.016	0
AFF3	1.059135042	0.557	0.303	3E-56
PRF1	1.0557477	0.525	0.153	6.2E-207
FCER1G	1.038095226	0.745	0.158	0
AUTS2	1.031436195	0.722	0.344	1.4E-135
HSH2D	1.02946759	0.513	0.198	3.5E-137
DUSP2	1.025463604	0.738	0.478	9.62E-82
TOX2	1.022758865	0.378	0.135	5.8E-103
ID2	1.019926404	0.752	0.344	9.7E-155
KRT81	1.012558224	0.272	0.001	0
CTNNB1	1.007312646	0.686	0.452	5.43E-85
CSF2	0.990504981	0.123	0.005	0
IL4I1	0.978441807	0.22	0.06	1.98E-86
HIP1	0.972476769	0.574	0.22	9.3E-145
IFITM1	0.971708283	0.618	0.296	3.4E-110
DHRS3	0.969596375	0.499	0.115	7.1E-280
RUNX3	0.968352475	0.823	0.522	2.1E-110
MAFF	0.962535116	0.52	0.148	1.4E-221
PPP1R9A	0.961773312	0.307	0.019	0
PRKX	0.958532683	0.682	0.388	2.4E-94
ARHGAP31	0.954514632	0.346	0.128	2.11E-88
PLXNA4	0.945420109	0.247	0.019	0
BHLHE40	0.936639018	0.566	0.197	3.4E-178
PDGFD	0.936143798	0.284	0.068	1.3E-135
SLA2	0.933137742	0.456	0.174	6.9E-113
TGFB1	0.930739379	0.84	0.604	2.9E-93
KIR2DL4	0.929336937	0.282	0.013	0
RALGAPA1	0.919466523	0.755	0.563	7.7E-59
ABCB1	0.910437	0.486	0.119	3.7E-232
TYROBP	0.905282257	0.837	0.199	0
ABHD2	0.89663553	0.493	0.174	2.2E-150
RAMP1	0.894464872	0.309	0.024	0
CEMIP2	0.891699671	0.726	0.574	4.67E-30
FES	0.887313369	0.375	0.057	0
PRMT9	0.88558194	0.514	0.283	1.13E-63
CD96	0.8833988	0.861	0.524	1.3E-102
HOPX	0.879081767	0.514	0.162	1.4E-168
CTBP2	0.872660848	0.406	0.104	1.5E-187
PLEKHA2	0.871380545	0.719	0.434	7.42E-97

FC, fold change; Pct.1, the expression level of selected gene in the current cluster; Pct.2, the expression level of selected gene in other clusters; P_val_adj, adjusted P value

Table S6 The marker genes and the panel used in mass cytometry (CyTOF).

List	Label	Marker	Clone
1	89Y	CD45	HI30
2	115In	CD3	UCHT1
3	139La	CD68	Y1/82A
4	141Pr	CD56	NCAM16.2
5	142Nd	gdTCR	5A6.E9
6	143Nd	CD196(CCR6)	G034E3
7	144Nd	CD38	HIT2
8	145Nd	CD45RA	HI100
9	146Nd	CD123	6H6
10	147Sm	CD183(CXCR3)	G025H7
11	148Nd	CD19	HIB19
12	149Sm	CD25	24212
13	150Nd	CD11c	BU15
14	151Eu	CD278(ICOS)	C398.4A
15	152Sm	CD195(CCR5)	J418F5
16	153Eu	CD161	HP-3G10
17	154Sm	CD294(CRTH-2)	BM16
18	155Gd	CD206	43876
19	156Gd	CD204	351615
20	157Gd	CD39	A1
21	158Gd	CD197(CCR7)	G043H7
22	159Tb	CD45RO	OCHL1
23	160Gd	CD14	M5E2
24	161dy	CD152(CTLA-4)	14D3
25	162Dy	FoxP3	PCH101
26	163Dy	CD163	GHI/61
27	164Dy	RORγt	600214
28	165Ho	CD66b	G10F5
29	166Er	CD117(c-kit)	104D2
30	167Er	KLRG1(MAFA)	SA231A2
31	168Er	T-bet	4B10
32	169Tm	CD27	O323
33	170Er	CD127	A019D5
34	171Yb	GATA3	TWAJ
35	172Yb	CD336(NKp44)	P44-8
36	173Y	GranzhymeB	GB11
37	174Yb	CD279(PD-1)	EH12.2H7
38	175Lu	CD16	3G8
39	176Yb	HLA-DR	L243
40	197Au	CD4	RPA-T4
41	198Pt	CD8	RPA-T8
42	209Bi	CD11b	ICRF44

Table S7 The basic sequencing parameters of six samples for spatial transcriptomics.

Items	IC 5	IC 10	IC 13	SUI 1	SUI 6	SUI 8
RQN value	7.75	7.82	7.50	7.51	7.54	8.5
Barcode Count in Whitelist	4992	4992	4992	4992	4992	4992
Detected Barcode Count	4992	4992	4992	4992	4992	4992
Detected Barcode UMI Count	17950566	12950662	12706213	8108471	4296068	7934005
Estimated Barcode Count	2709	2551	2235	1793	1583	3074
Estimated Barcode UMI Count	15447752	10921548	10243352	6164150	3326952	7010338
Mean Reads per Spot	205984	240809	267872	407612	399428	197023
Median UMI Counts per Spot	3987	3067	3009	2862	1365	2045
Sequencing Saturation	96.00%	97.40%	97.40%	98.30%	99.00%	98.30%
Total Genes Detected	21910	20844	20869	19154	18587	20733
Median Genes per Spot	2000	1685	1559	1442	792	1049

IC, interstitial cystitis; UMI, unique molecular identifiers.
