# Supplementary figures and images for: Integrating single-cell RNA sequencing with spatial transcriptomics reveals immune landscape for interstitial cystitis
Source: Signal Transduct Target Ther. 2022 May 20;7:161. doi: 10.1038/s41392-022-00962-8 (PMC9120182; doi:10.1038/s41392-022-00962-8)

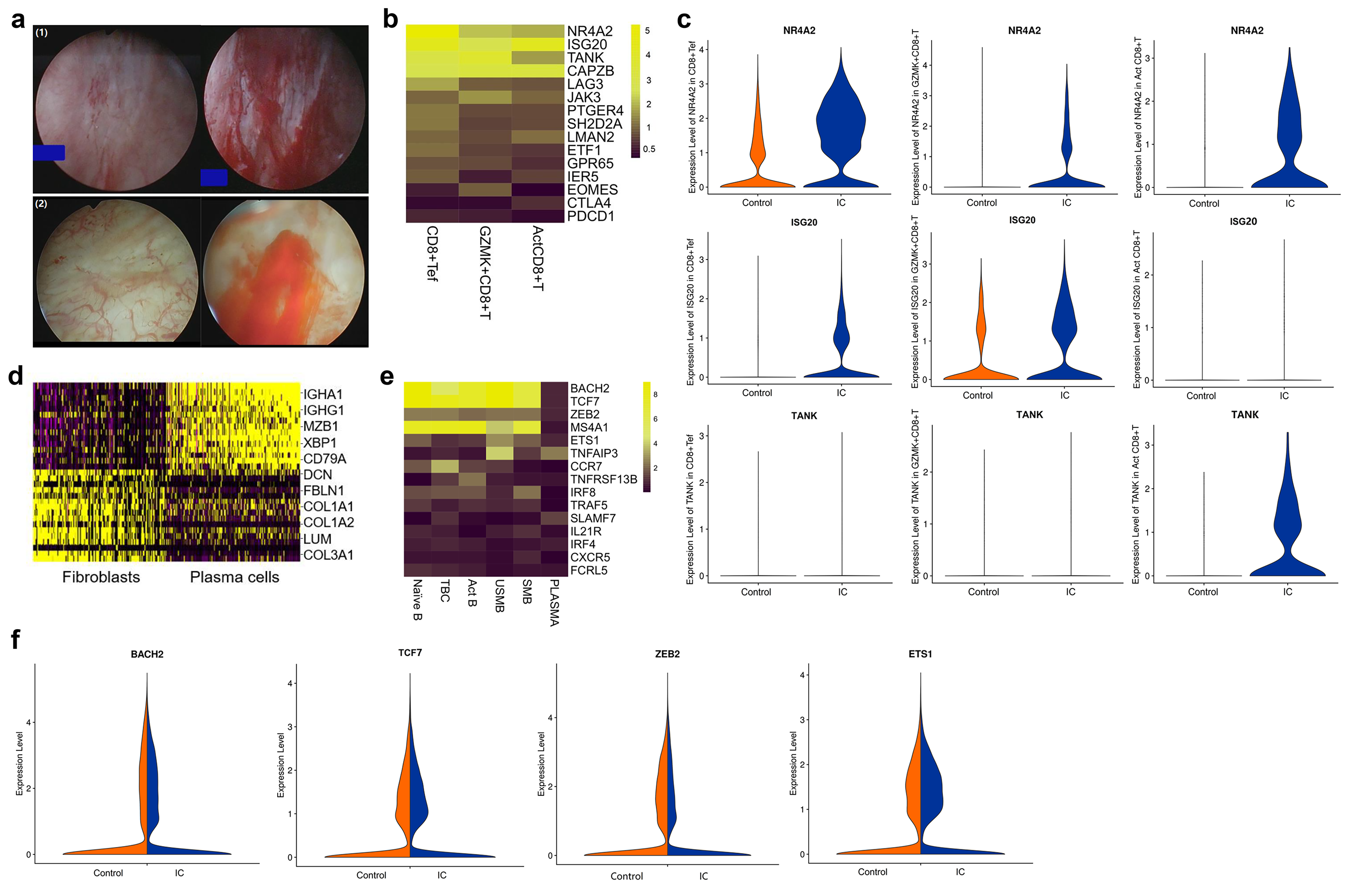

Supplement: Supplementary file 2 — Supplementary Fig. 1 [file 41392_2022_962_MOESM2_ESM.tif]

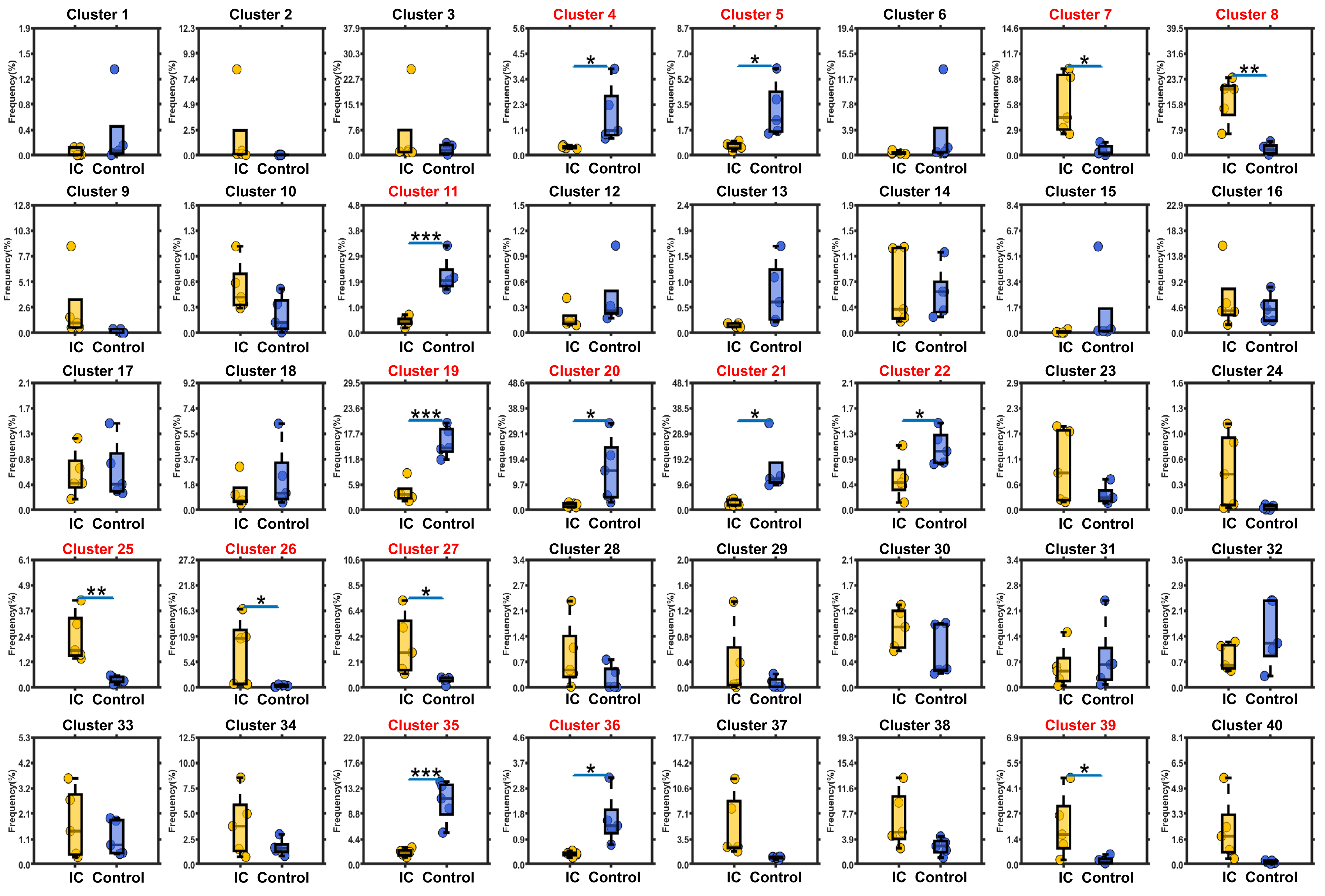

Supplement: Supplementary file 3 — Supplementary Fig. 2 [file 41392_2022_962_MOESM3_ESM.tif]

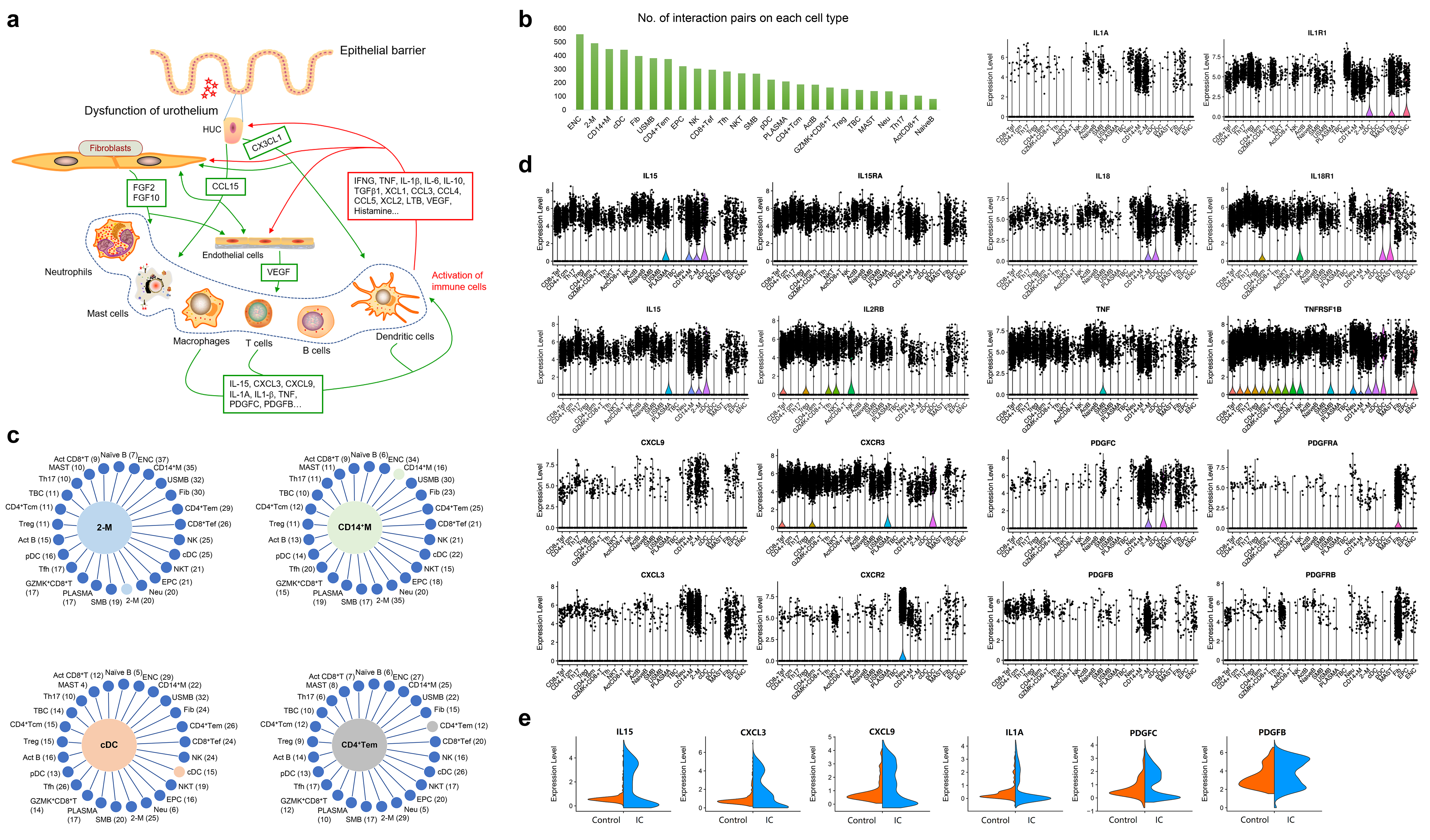

Supplement: Supplementary file 4 — Supplementary Fig. 3 [file 41392_2022_962_MOESM4_ESM.tif]

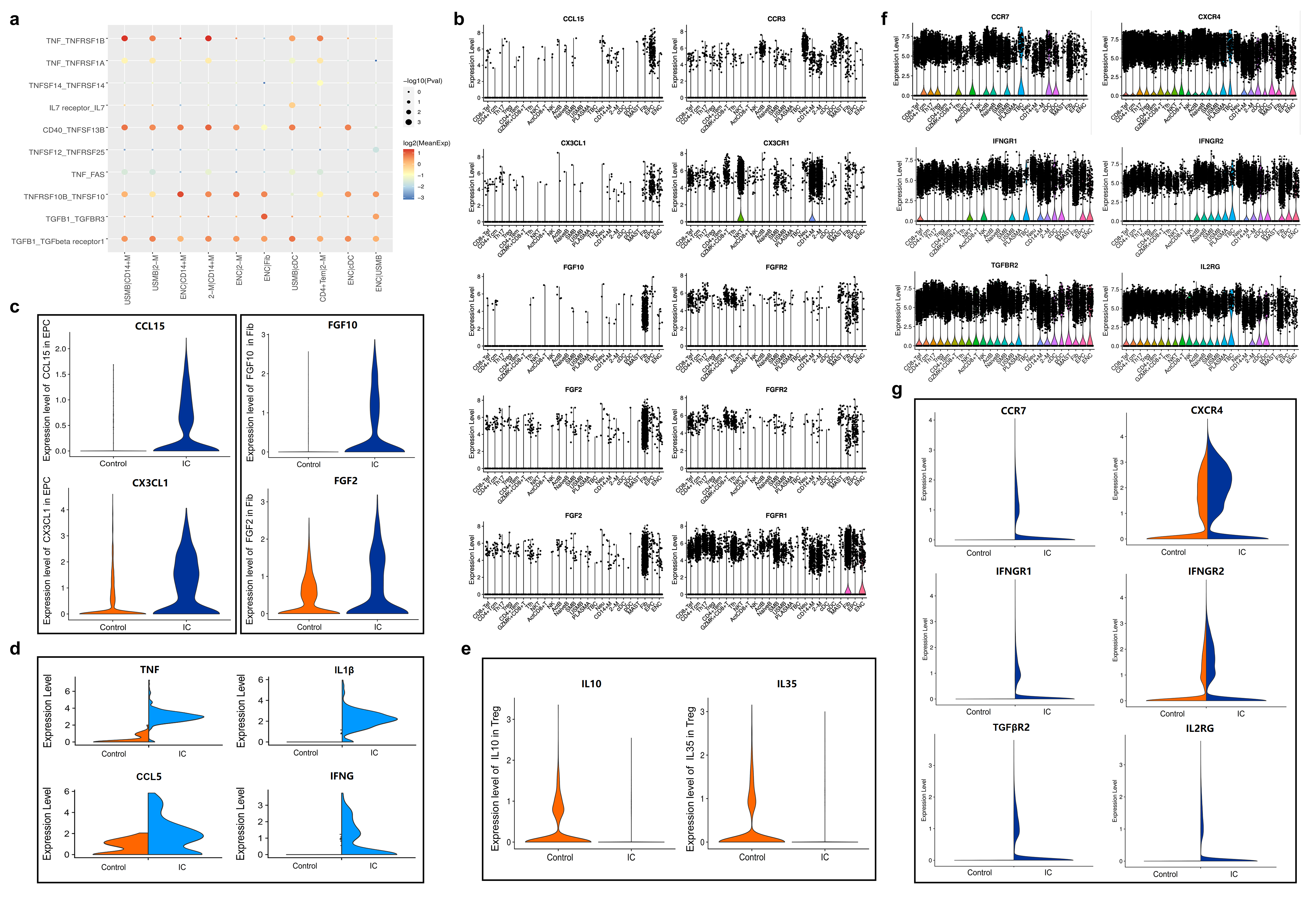

Supplement: Supplementary file 5 — Supplementary Fig. 4 [file 41392_2022_962_MOESM5_ESM.tif]

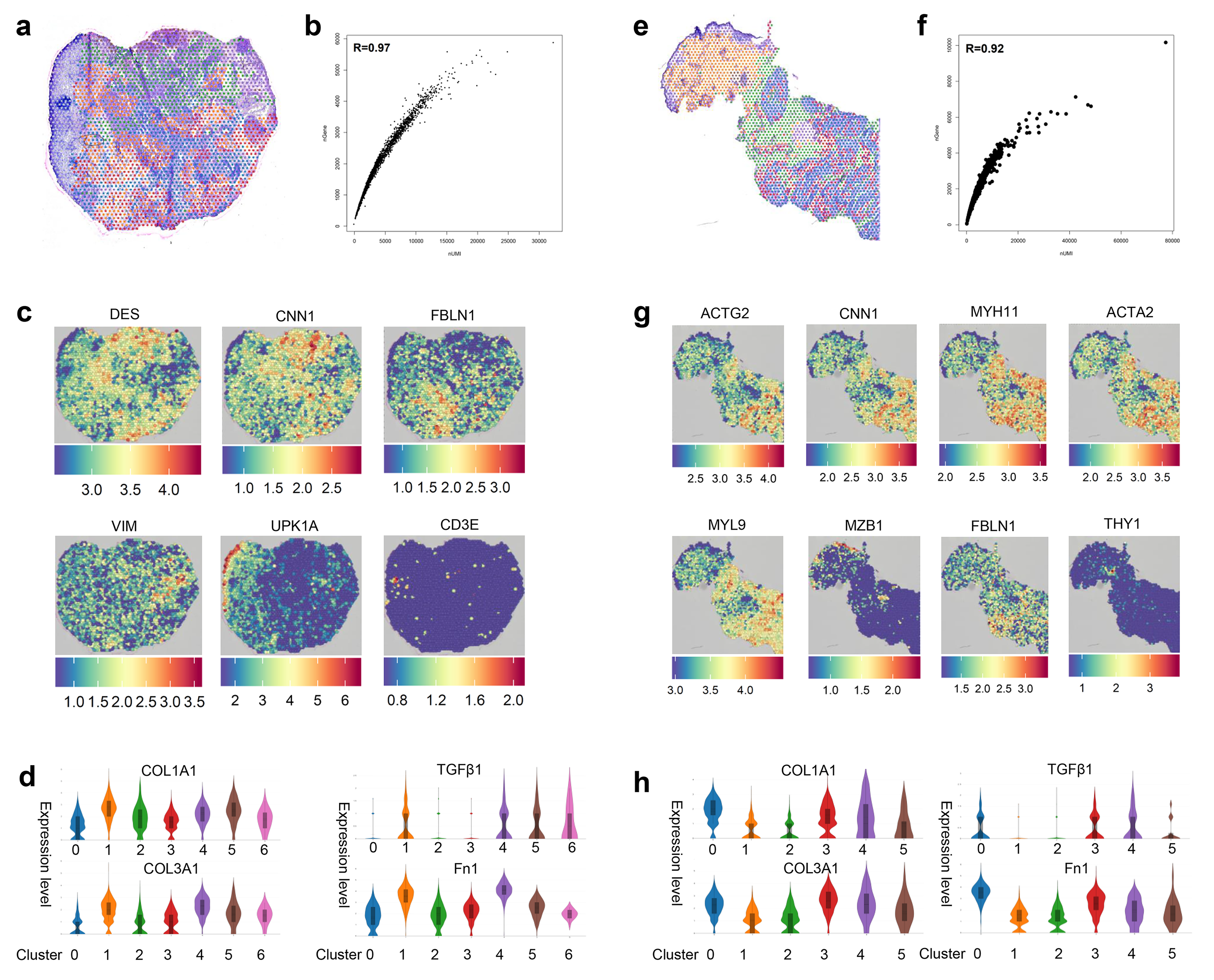

Supplement: Supplementary file 6 — Supplementary Fig. 5 [file 41392_2022_962_MOESM6_ESM.tif]

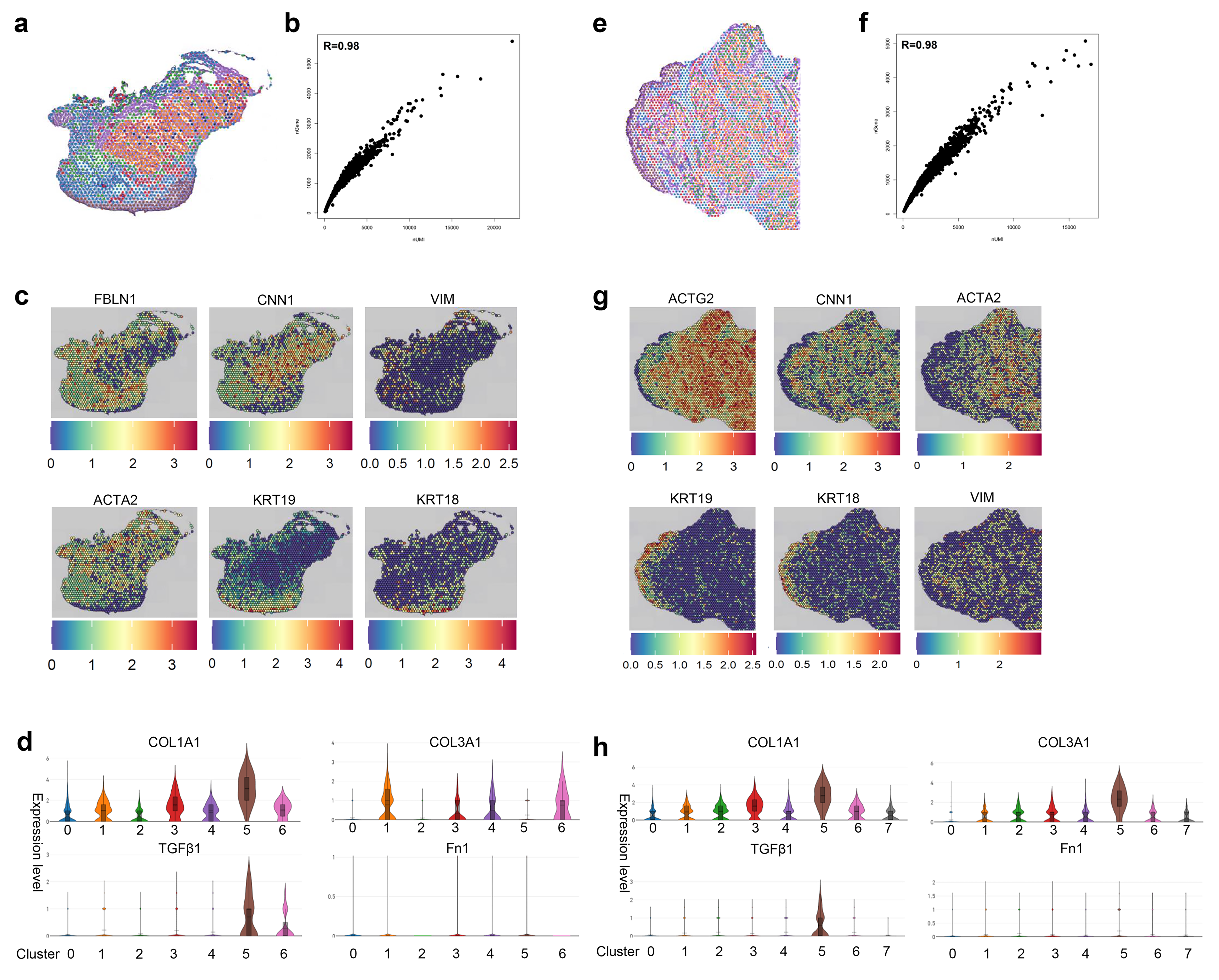

Supplement: Supplementary file 7 — Supplementary Fig. 6 [file 41392_2022_962_MOESM7_ESM.tif]

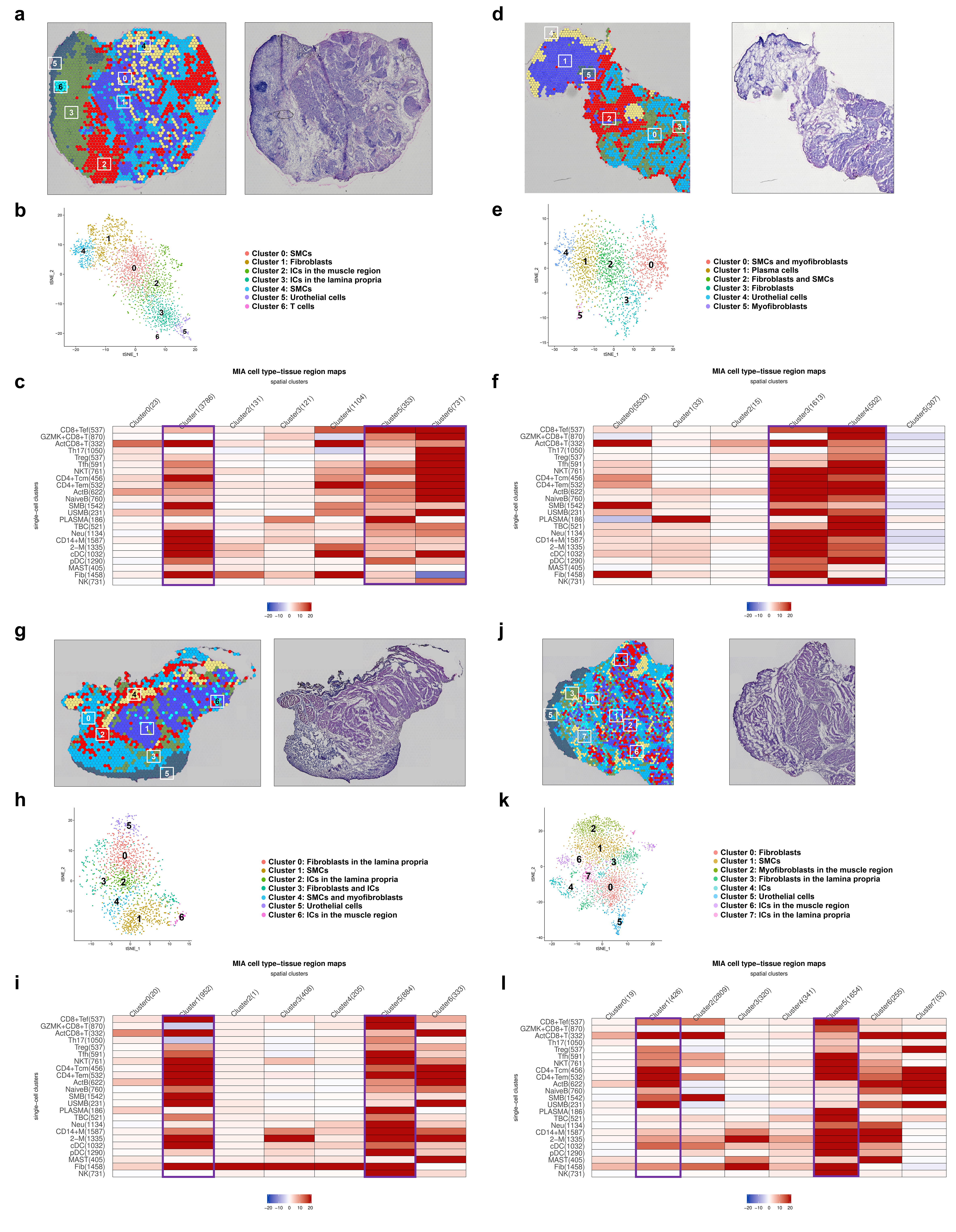

Supplement: Supplementary file 8 — Supplementary Fig. 7 [file 41392_2022_962_MOESM8_ESM.tif]
